# How does high temperature weather affect tourists’ nature landscape perception and emotions? A machine learning analysis of Wuyishan City, China

**DOI:** 10.1371/journal.pone.0323566

**Published:** 2025-05-15

**Authors:** Cuicui Ye, Zhengyan Chen, Zheng Ding

**Affiliations:** 1 College of Art, Wuyi University, Mount Wuyi, Fujian, China; 2 College of Arts College of Landscape Architecture, Fujian Agriculture and Forestry University, Fuzhou, Fujian, China; Zhejiang Agriculture and Forestry University: Zhejiang A and F University, CHINA

## Abstract

Natural landscapes are crucial resources for enhancing visitor experiences in ecotourism destinations. Previous research indicates that high temperatures may impact tourists’ perception of landscapes and emotions. Still, the potential value of natural landscape perception in regulating tourists’ emotions under high-temperature conditions remains unclear. In this study, we employed machine learning models such as LSTM-CNN, Hrnet, and XGBoost, combined with hotspot analysis and SHAP methods, to compare and reveal the potential impacts of natural landscape elements on tourists’ emotions under different temperature conditions. The results indicate: (1) Emotion prediction and spatial analysis reveal a significant increase in the proportion of negative emotions under high-temperature conditions, reaching 30.1%, with negative emotion hotspots concentrated in the downtown area, whereas, under non-high temperature conditions, negative emotions accounted for 14.1%, with a more uniform spatial distribution. (2) Under non-high temperature conditions, the four most influential factors on tourists’ emotions were Color complexity (0.73), Visual entropy (0.71), Greenness (0.68), and Aquatic rate (0.6). In contrast, under high-temperature conditions, the most influential factors were Greenness (0.6), Openness (0.56), Visual entropy (0.55), and Color complexity (0.55). (3) Compared to non-high temperature conditions, high temperatures enhanced the positive effects of environmental perception on emotions, with Greenness (0.94), Color complexity (0.84), and Enclosure (0.71) showing stable positive impacts. Additionally, aquatic elements under high-temperature conditions had a significant emotional regulation effect (contribution of 1.05), effectively improving the overall visitor experience. This study provides a data foundation for optimizing natural landscapes in ecotourism destinations, integrating the advantages of various machine learning methods, and proposing a framework for data collection, comparison, and evaluation of natural landscape perception under different temperature conditions. It thoroughly explores the potential of natural landscapes to enhance visitor experiences under various temperature conditions and provides sustainable planning recommendations for the sustainable conservation of natural ecosystems and ecotourism.

## 1. Introduction

With the continuous advancement of global urbanization, natural experiences and ecotourism are playing an increasingly significant role in promoting human health and well-being [1]. Ecotourism not only adds value to the daily lives of residents and tourists but also brings sustainable economic benefits [2]. It has a positive and significant long-term impact on national green economic growth, creating a virtuous cycle from the enhancement of natural ecological value to tourism economic development and ultimately to improving human well-being. However, in recent years, due to global climate warming, many regions worldwide, especially in Europe, Australia, and large parts of Asia, have experienced frequent and intense extreme high-temperature events [3]. These unstable high-temperature events have led to environmental degradation, increased health risks, and reduced human well-being [4], significantly impacting tourists’ landscape perception and emotional experiences in ecotourism. Emotional changes caused by high temperatures, environmental, or individual factors during tourism activities directly affect tourists’ motivations, satisfaction, and behavioral intentions [5]. This can hinder the sustainable growth of green tourism economies and improve quality of life. In the context of sustainable planning for ecotourism, exploring how high temperatures and temperature changes affect tourists’ landscape perception and emotions helps planners and practitioners understand tourists’ preferences for landscape perception and emotional characteristics under different temperature conditions. This knowledge can be used to develop adaptive ecotourism strategies and differentiated natural landscape protection plans. Such efforts are crucial for the sustainable planning of global natural resources and ecotourism development. Additionally, they contribute to achieving the UNWTO’s goals for sustainable tourism and “sustainable consumption and production,” as well as the United Nations’ goal of ensuring healthy lifestyles and promoting well-being for all ages[6].

As global temperatures approach an increase of 3°C, scholars have increasingly begun to explore the far-reaching impacts of climate change and mitigation measures on human well-being [7,8]. They have focused mainly on the negative effects of high temperatures, such as the impact on infectious disease prevalence [9], the influence on cardiovascular diseases [10], emotional deterioration [11], and the psychological health issues caused by high temperatures [12]. In the fields of landscape and ecology, most studies have focused on the macro-level impacts of climate change and high temperatures, such as landscape sensitivity [13], landscape ecological risk prediction [14], landscape structural changes [15], ecosystem services [16] and the interaction perspective of thermal radiation, human, activity, and space [17]. Although numerous studies have demonstrated that temperature and environment affect human activities, emotions, perception, and thermal comfort in outdoor spaces [18–20] and influence tourists’ behavioral intentions and aesthetic experiences in ecotourism [21], the mechanisms underlying how temperature changes affect tourists’ micro-level visual preferences and emotional landscape perception remain unclear. Specifically, gaps exist in understanding tourists’ landscape perception differences and emotional fluctuations under varying temperature conditions. Furthermore, most existing studies pay little attention to the interactions between tourists’ emotional perceptions and natural landscapes under high temperatures and the nonlinear associations of these interactions under different temperature conditions.

In recent years, a large number of researchers studying ecotourism and tourists’ natural landscape perception have employed traditional methods such as surveys [22,23], interviews [24], and case studies [25] to explore the impact of natural landscapes from different perspectives. However, there is a lack of an efficient framework for obtaining data on landscape perception, and the disorderliness, diversity, and randomness of recreational behavior in ecotourism make data collection challenging. Consequently, using surveys, psychological experiments, and other subjective emotional analysis approaches to analyze large-scale, multi-objective targets is challenging. Most research has focused on the impact of natural landscape perception on tourists’ physical and mental health [26,27], including aesthetic preferences for micro-landscape features [28], perception effects of blue-green spaces [29], the impact of natural landscapes on tourists’ emotions [30], Influence of open space morphology and tree canopy on comfort levels [31] and tourists’ satisfaction with natural landscape functions [32]. These studies further confirm the importance of natural landscapes in promoting physical and mental health and well-being, revealing the positive benefits of natural landscapes and providing theoretical support for ecotourism planning practices. While most studies have explored the positive effects of natural landscapes from a macro perspective in the context of ecotourism, few have investigated the contribution of specific landscape features to positive and negative emotional changes. Moreover, seasonal and climatic changes significantly affect the state and perception of natural landscapes [33]. However, few studies have examined the impact of natural landscapes on tourists’ emotions under different weather and temporal conditions.

The development of machine learning methods and social media platforms has opened new avenues for research into ecotourism and tourists’ emotional perception of landscapes, significantly enhancing data acquisition efficiency and complex indicator analysis. Building on this, numerous studies have utilized machine learning techniques combined with user-generated content (UGC) to analyze the coupling relationship between natural landscape perception and human emotions in various contexts, employing models such as Deepsentibank [34], ResNet [35], Inception-v3 [36], XGBoost [37], and Word2vec [38]. However, prior research often analyzes geotagged UGC text data parallel to macro-level remote sensing big data. Due to the diverse and unstable nature of emotional components in UGC text information [39], this approach may lead to mismatches between actual landscape data and emotion data quantified from text. Moreover, standardized evaluation systems for utilizing big data are still underdeveloped; macro data such as land use distribution, natural vegetation status, and other remote sensing images are often limited by a lack of detailed information [40]. This limitation is especially pronounced in ecotourism activities that focus on natural landscape perception, where the absence of visual perception data often makes it challenging to quantify micro-level indicators. Owing to data constraints, most studies employ broad-scale remote sensing or landscape big data to quantify the overall impact of the environment on residents’ emotions, potentially overlooking the collinearity and interdependencies among various indicators. Regarding research subjects, current studies predominantly focus on urban tourism and the emotional perception of built environments [41–43], with less attention paid to the mechanisms of natural landscape perception benefits under climate change. Additionally, the “black box” effect during the training process of machine learning methods [44] limits the interpretability of these models. Therefore, an urgent need is to develop an evaluation framework that can accurately assess and compare natural landscape perceptions under different climatic conditions. This would enable designers and managers to utilize landscape big data better, thereby enhancing the efficiency of natural ecological resource conservation and use and improving the practicability of current experimental methods and results.

The development of ecotourism and sustainable conservation of natural landscapes is of significant importance to advancing the United Nations Sustainable Development Goals (SDGs) [45]. Mount Wuyi is one of China’s premier national scenic spots, boasting rich and representative natural ecological resources. In December 1999, Mount Wuyi was inscribed on the UNESCO World Heritage List (No. 911) as a site of cultural and natural significance, meeting the World Heritage selection criteria (III, VI, VII, and X). Since then, it has become one of China’s four cultural and natural heritage sites, exemplifying generations of harmonious coexistence between humans and nature [46]. Under this heritage influence, tourism dominates the economic system of Wuyishan City, with its development of ecotourism, natural resource exploitation, and recreational use being highly representative. From 2019 to 2023, the average annual number of tourists to Mount Wuyi National Park alone was 12.32 million (data from the Statistical Yearbook of Wuyishan City, Fujian Province) [47]. Wuyishan City and its province, Fujian, are significantly affected by extreme high-temperature weather, including large-scale persistent heat anomalies. For instance, the highest temperature on July 24, 2022, reached 41.9°C, setting a record since 1961 [48]. Climate change and the increasing frequency of extreme high-temperature events challenge ecotourism development in Wuyishan City. Therefore, in-depth research into the rich natural landscapes of Wuyishan City and the exploration of their utilizable value are of great significance for the development of natural ecological resources worldwide. However, existing research focusing on the natural landscapes of Mount Wuyi mainly centers on case studies of representative sites like Mount Wuyi National Park [49–51]. With the gradual development of ecotourism and natural landscape recreational resources in Wuyishan City, current research should not be confined to single case studies centered on national parks. Instead, it should broaden its perspective to construct an evaluation framework for the overall perception of the natural landscape of Wuyishan City. Additionally, in line with China’s “14th Five-Year Plan” for tourism development—which emphasizes a shift from low-level to high-quality and diversified consumer demand—it is essential to reasonably advance the development of green ecotourism.

Based on this, our experiment focuses on points of interest (POI) in the natural landscapes of Wuyishan City, China, as the research subjects. By comparing the differences in tourists’ landscape and emotional perceptions under high-temperature versus non-high-temperature conditions, we aim to address two significant research gaps. First, current studies fail to examine how high-temperature conditions influence tourists’ perceptions of natural landscapes, lacking systematic analyses of the variations in landscape benefits under different temperature scenarios, particularly concerning the intricate effects of specific landscape configurations on human emotions. Second, due to limitations in current data processing methods and quantification frameworks, existing research on evaluating natural landscape perceptions lacks an efficient, precise, and highly applicable assessment model. Building on this, we further propose the objectives of this study: (1) To fully leverage social media platforms and user-generated content, including official daily climate data from China Meteorological Data Service Center (CMA), while combining the unique advantages of various machine learning models and geographic analysis techniques to explore how high-temperature conditions affect tourists’ landscape perceptions and emotions. (2) To investigate the complex nonlinear relationships and interactions between natural landscape perception and tourists’ emotions using a framework that integrates multiple disciplinary methods, including various machine learning approaches, thereby providing a data foundation for related research on natural landscape perception and the emotional benefits of tourists’ experiences with landscapes. (3) Based on the experimental results, to propose sustainable planning and design recommendations for the natural landscape resources of Wuyishan City, China, and to offer valuable insights for the sustainable development of green ecotourism worldwide through differentiated ecotourism and natural landscape resource planning methods.

## 2. Data and methodology

### 2.1. Study area

**Fig 1:**
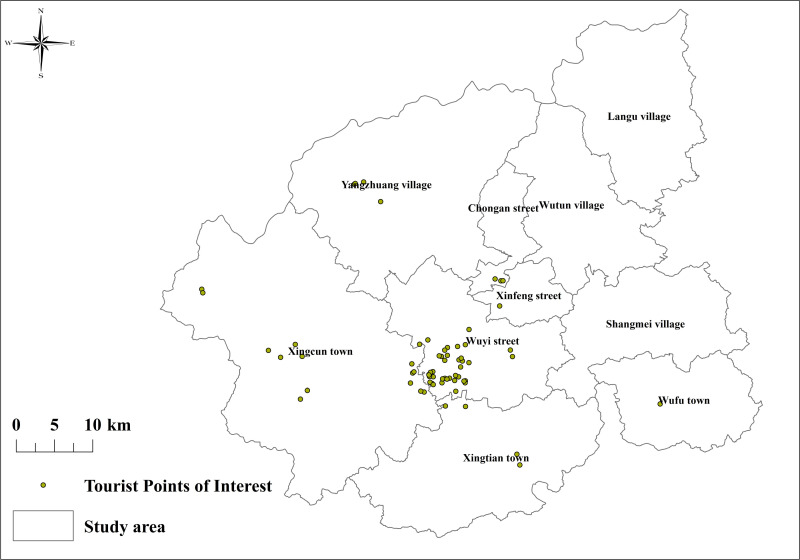
Study area Note: Based on the standard map production of the National Natural Resources Department’s Standard Map Service website GS(2019) 1822, the base map boundaries have not been modified. (http://bzdt.ch.mnr.gov.cn/download.html?searchText=GS(2019)1822).

Wuyishan City spans a total area of 2,813.91 square kilometers and is situated near the Tropic of Cancer (longitude 117°37′ to 118°19′ E, latitude 27°27′ to 28°05′ N). The city is positioned in a subtropical region known for a typical subtropical monsoon climate with notable regional climatic variations. The annual average temperature is 18.3 °C, relative humidity fluctuates between 78% and 84%, and annual precipitation totals 1,888.1 mm, mostly occurring from April to June. The average annual sunshine duration reaches approximately 1,910.2 hours. [52,53]. These climatic characteristics make Wuyishan City an ideal case for comparing natural landscape perceptions under high-temperature conditions. The consistently stable climate provides a rich data foundation for comparative studies under different temperature conditions, aiding in understanding tourists’ perceptual and emotional changes under high temperatures and assisting planners in designing adaptive tourism experiences. The study area is shown in Fig 1, where we identified 80 tourist POI with significant visitor activity from social media platforms and mapped their distribution.

### 2.2. Technical route

**Fig 2:**
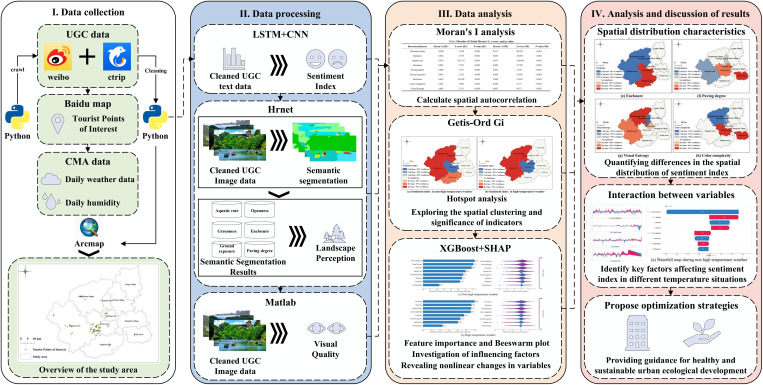
Technical route Note: Based on the standard map production of the National Natural Resources Department’s Standard Map Service website GS(2019) 1822, the base map boundaries have not been modified. (http://bzdt.ch.mnr.gov.cn/download.html?searchText=GS(2019)1822).

To illustrate the complex relationships among the relevant feasibilities and to present a comprehensive framework for this study, the technical route employed in the experiment is presented in Fig 2. Additionally, to effectively compare the potential impacts of climate change under high-temperature versus non-high-temperature conditions on tourists’ experiences of natural landscapes, relevant user-generated content (UGC) comprising both images and texts was collected from social media platforms. We integrated the unique advantages of various machine learning methods and proposed an emotion analysis framework centered around the Long Short-Term Memory-Convolutional Neural Network (LSTM-CNN) model. The LSTM network’s long-term memory capabilities and the CNN’s local feature extraction abilities were utilized to process text data, enhancing model performance through their combined strengths. High-Resolution Net (Hrnet) and MATLAB ensured the quality of detail processing in landscape image quantification through high-resolution and compatible processing methods. Additionally, ArcMap 10.8 software ensured the visibility and readability of the results, with its spatial autocorrelation analysis and hotspot analysis verifying the relationships between different variables and spatial autocorrelation. Finally, we used Extreme Gradient Boosting (XGBoost) and SHapley Additive exPlanation (SHAP) to analyze the reasons behind differences in tourists’ perceptions under different temperature conditions and to explain the contribution and interactions of other variables to the model. The experimental process, shown in Fig 2, is divided into three steps: data collection, indicator computation, and data analysis.

(1) Data collection: Social media data were obtained from Weibo (https://weibo.com/) and Ctrip (https://you.ctrip.com/). We input relevant keywords related to eco-tourism in Wuyishan City using web crawling techniques while retrieving information. We removed advertisements, newspaper promotions, invalid image links, and non-landscape or invalid photo data. Ultimately, we organized 57,281 relevant UGC entries containing natural landscape photos and text comments about eco-tourism. We identified 80 eco-tourism POIs with significant tourist activity using geographic information software. All data were collected within the past five years (July 2019 to July 2024).
(2) Indicator computation: After pre-training, the LSTM-CNN model was used for sentiment analysis of UGC texts. Hrnet and MATLAB were employed to extract visual quality indicators and landscape perception indicators from images. Spatial autocorrelation validation and hotspot analysis were performed using ArcMap software for result visualization.
(3) Data analysis: We used the organized dataset for XGBoost modeling and SHAP analysis, calculating the marginal effect values and contributions of different natural landscape variables on tourists’ emotions. We employed SHAP feature importance analysis, SHAP partial dependence plots, SHAP waterfall plots, and SHAP force plots in the experiment. We compared different natural landscape perception indicators under non-high-temperature and high-temperature conditions, including differences in tourists’ landscape perception emotions and the nonlinear associations of different indicators with emotional perception.

### 2.3. Selection of variables

Tourists’ satisfaction with the environment is closely related to their experience of natural eco-tourism, and approximately 76% of people’s environmental satisfaction is associated with visual elements [54]. Therefore, constructing a research framework that evaluates landscape quality while considering aesthetic preferences is significant for managing natural landscape resources [55]. To ensure the objectivity of experimental indicator selection and facilitate better discussion of our results with peer research, we reviewed and organized significant publications related to this topic from 2014 to 2024. We screened quantitative indicators of natural landscape emotion perception used in these publications and supplemented them based on the innovative aspects of our experiment. Combining insights from previous studies on natural landscape preferences [56] and preliminary analysis of natural landscape photos before the experiment, we categorized natural landscape element indicators into three main groups: natural elements, artificial features, and visual characteristics.

Understanding the diverse values and perceptions associated with elements of natural landscapes is essential, as a reciprocal relationship exists between people and landscapes; how landscapes shape human perceptions may, in turn, influence people’s behaviors and actions within landscape settings [57]. Existing research often considers the openness and diversity of landscapes when exploring natural landscape perception elements. For example, mountains and sky represent openness, and artificial pavement and the degree of exposure to natural soil are important indicators affecting tourists’ experiences [58–60]. Nevertheless, green vegetation such as trees, shrubs, grass, exposed soil, building enclosure, and aquatic rate are critical indicators for quantifying natural landscape configurations [61]. The visibility of green space has been extensively studied to explore the impact of natural landscapes on human emotional health [62,63]. Consequently, as suggested by previous studies, it is necessary to examine further how the proportions of vegetation and water elements within landscapes affect tourists’ perceptions under varying temperature scenarios. Finally, regarding visual characteristics, existing studies have typically focused only on extracting objective visual elements or subjective perceptions, while overlooking the comprehensive role of the overall visual quality of landscapes [64]. Natural landscapes’ complexity and visual richness affect the overall level of landscape perception [43], but few studies have deeply explored the complex causes of these indicators.

Based on this, we rigorously screened the existing photographic data according to the established selection criteria for natural landscapes, excluding images unrelated to natural landscapes in eco-tourism. Table 1, “Research Element Breakdown,” presents the indicators and descriptions selected in this study. The dependent variable, the Sentiment Index, represents tourists’ emotion scores predicted by the LSTM-CNN framework. We selected independent variable indicators for different research elements by organizing and comparing previous research designs. We chose Openness, Greenness, and Enclosure for landscape element indicators to quantify common plant elements, sky elements, and environmental enclosure elements in natural landscape configurations. We used the paving degree and aquatic rate to calculate land exposure and water body elements in landscape configurations that might affect perception; these elements were quantified using the Hrnet model. Finally, to objectively assess tourists’ visual experiences in natural landscapes, we included visual evaluation indicators such as Visual Entropy and Color Complexity, calculated using Matlab, to determine the visual quality in landscape configurations.

**Table 1:** Research Element Breakdown Table.

Research Elements	Research indicators	Indicator Description	Quantitative methods
**Tourist emotions**	Sentiment index	NLP text data sentiment scoring	LSTM-CNN
**Landscape elements**	Openness	The proportion of sky in the image	Hrnet
**Landscape elements**	Aquatic rate	The proportion of water bodies in the image	Hrnet
**Landscape elements**	Greenness	The proportion of green plants in the image	Hrnet
**Landscape elements**	Paving degree	The proportion of floor covering in the image	Hrnet
**Landscape elements**	Ground exposure	Soil exposure index in the environment	Hrnet
**Landscape elements**	Enclosure	The proportion of architectural enclosure in the image	Hrnet
**Visual Quality**	Color complexity	Color complexity of images	Matlab
**Visual Quality**	Visual Entropy	Entropy value of images	Matlab

### 2.4. Data collection and processing

This study strictly adheres to the terms of use and privacy policies of the data source platforms when collecting and utilizing social media data. Only publicly available and non-private content was analyzed, ensuring that no personally sensitive information was collected. All user identifiers were anonymized, and the findings are solely for academic purposes. Furthermore, this study did not share any raw data with third parties, ensuring that user privacy remains fully protected.

#### 2.4.1. Daily climate data.

Daily climate data were obtained from the China Meteorological Data Service Center (http://data.cma.cn), a highly authoritative and reliable platform. The data have undergone strict monitoring and review to ensure accuracy and scientific validity, providing daily climate data that meet the experimental requirements. To ensure the rigor of the experiment, we distinguished high-temperature weather from non-high-temperature weather based on daily temperatures. The criteria for distinguishing high-temperature weather refer to the studies by Xu et al. [65] and Yao et al. [66] and China’s recommendations for triggering high-temperature warnings. Days with a maximum daily temperature greater than or equal to 35 °C were defined as high-temperature weather. Humidity is a crucial factor affecting comfort and perception [67] and can exacerbate the association between temperature and human mental health [68]. Therefore, to screen the data, we set average climate thresholds, such as relative humidity between 78% and 84% and no precipitation on the day. This ensures an accurate assessment of the impact of temperature changes on tourists’ natural landscape and emotional perception. The data collected were rigorously cleaned according to the experimental requirements established above.

#### 2.4.2 User generated content (UGC) datasets.

According to research needs, the social media UGC data in this study were obtained from Weibo (https://weibo.com/), Dianping (https://www.dianping.com/), and Ctrip (https://you.ctrip.com/). Using web crawling techniques, we input relevant keywords for attractions in Wuyishan City and collected image-containing comments within the past five years (July 2019 to July 2024). The data include post ID, text information, corresponding image URLs, and the number of shares, ensuring that natural landscape photos and comments used for sentiment analysis are matched. This addresses the research gap of mismatched image and text data in the current field. After collection and organization, we removed advertisements, newspaper promotions, invalid image links, and photos not related to landscapes or built environments to ensure the accuracy and objectivity of the experimental results. In total, 57281 UGC entries were collected during the experiment. After data collection and preprocessing, entries containing advertisements, promotional content from newspapers and magazines, invalid image links, and photos depicting non-landscape or built environments were removed to ensure the accuracy and objectivity of the experimental results. Finally, according to the experiment’s predefined temperature and humidity thresholds, 18237 relevant eco-tourism entries containing photographs of natural landscapes along with textual comments were identified, from which a parallel UGC dataset integrating visual and textual data was constructed.

#### 2.4.3. Long Short Term Memory networks-Convolutional Neural Networks (LSTM-CNN).

The LSTM-CNN framework leverages LSTM’s long-term memory capabilities and CNN’s local feature extraction abilities when processing text data. It significantly enhances model performance by combining their advantages to meet the requirements for analyzing tourists’ emotional texts [69]. The pre-trained model used in the experiment is ERNIE (https://github.com/PaddlePaddle/LARK/tree/develop/ERNIE). After constructing the UGC dataset with synchronized images and texts, we used the LSTM-CNN method to score the texts using the trained Natural Language Processing (NLP) model. Scores ranged from -10–10, where higher values represent stronger positive emotions. The LSTM-CNN model achieved an overall accuracy of 95.4% in identifying complaint-related emotions, accompanied by a low test loss value of 0.12, signifying strong prediction capability and stability. Specifically, the precision, recall, and F1-score for positive emotion classification were 95.9%, 94.9%, and 95.4%, respectively, while for negative emotions, these metrics were 94.9%, 95.8%, and 95.3%, respectively. Such results highlight the model’s balanced and efficient performance distinguishing between positive and negative complaint emotions. Additionally, the model demonstrates robustness in addressing class imbalance, achieving high predictive precision and comprehensive coverage across emotional categories, thus fully satisfying the experimental criteria for accurate emotion recognition.

#### 2.4.4. High-Resolution Net (Hrnet) for Semantic Segmentation.

We utilized the Hrnet model, known for its strong generalization ability and stability, to calculate landscape element data in images. This framework improves the performance of semantic segmentation tasks by parallelly combining high- and low-resolution convolutions. Due to the varying resolutions and sizes of natural landscape images obtained from UGC data, we chose this network to address the challenge and ensure the accuracy of the results. The framework enhances high-resolution representations by aggregating upsampled parallel convolutions, achieving better results in image-processing tasks. On the Cityscapes test set, the model achieved mIoU (81.6%), iIoU (61.8%), cla. IoU (92.1%), and iIoU cat. (82.2%), demonstrating improvements over DenseASPP by 1.0%, 2.7%, and 4.1%, respectively, outperforming PSPNet by 3.2% in mIoU. Additionally, compared to ResNet38, the model showed a 1.2% improvement in cla. IoU. On the LIP dataset, it achieved a Pixel Accuracy of 88.21%, highlighting its strong segmentation performance across different datasets [70]. In the experiment, Hrnet extracted indicators such as Openness, Aquatic rate, Greenness, Paving degree, and Enclosure from images. The quantification methods for these landscape element indicators refer to the study by Wu et al. [71]. Table 2, titled “Semantic Segmentation Recognition Explanation,” presents the computation process for each landscape element indicator.

**Table 2:** Semantic segmentation recognition explanation.

Research indicators	Explanation
**Aquatic rate (A)**	A = P_Aquatic rate_/P_Total_*100%, A represents the final Aquatic rate of the image; P_Aquatic_ rate is the total pixel count of water elements identified by the model; P_Total_ is the total count of pixels recognized in the image.
**Openness (O)**	O = P_Sky_/P_Total_ *100%, O represents the final Openness value of the image; P_Sky_ is the total pixel count of sky elements identified by the model; P_Total_ represents the total recognized pixel count in the image.
**Greenness (G)**	G = P_Greenness_/P_Total_ *100%, G represents the percentage of ground covered by vegetation in the image; P_Greenness_ is the pixel count of grass and other green ground elements identified by the model; P_Total_ is the total recognized pixel count in the image.
**Enclosure (E)**	M = P_Enclosure degree_/P_Total_ *100%, M represents the percentage of Mountain elements in the image; P_Enclosure degree_ is the pixel count of mountain elements identified by the model; P_Total_ is the total recognized pixel count in the image.
**Ground exposure (G1)**	G_1_ = P_Ground exposure_/P_Total_ *100%, M represents the percentage of Soil exposure index in the image; P_Ground exposure_ is the pixel count of mountain elements identified by the model; P_Total_ is the total recognized pixel count in the image.
**Paving degree (P)**	P = P_Paving degree_/P_Total_ *100%, P represents the percentage of exposed soil in the image; P_Paving degree_ is the pixel count of exposed land identified by the model; P_Total_ is the total recognized pixel count in the image.

#### 2.4.5 MATLAB-based color complexity and visual entropy calculation.

Visual entropy is a key visual feature affecting the perception of landscapes at more minor scales. In fields such as landscape ecology, landscape aesthetics, and user visual perception, entropy is commonly used as an indicator to measure the overall complexity of images. Using MATLAB software, visual entropy was calculated through steps including image grayscale enhancement, region segmentation, and area calculation. The formula for this process is as follows [72]:


$$H(x)=-\sum_{i=1}^{n}P\left(a_{i}\right)*logP(a_{i})$$ (1)

*i* represents the divided region, whereas P(ai) denotes the probability of region ai’s occurrence (*i* = 1, 2,..., n). Additionally, *H(x)* indicates the total amount of information generated for the complete visual object composed of n regions. Additionally, *H(x)* denotes the total amount of data generated for the entire visual object composed from n areas. Further, *H(x)* indicates the total amount of information generated for the complete visible object composed of n regions.

Color in landscape configurations can affect human perception levels [73], and color complexity is an essential indicator for capturing color characteristics in images. The principle of color complexity evaluation is displayed in Equation 2 [74]:


$$C_{k}=-\sum_{i=1}^{m}n_{i}\log(\frac{n_{i}}{N}\backslash$$ (2)

Where *C*_*k*_ means the complexity of the spatial distribution feature of a particular color; *m* is the number of different connected regions in the general set; *n*_*i*_ is the number of pixels of the ith connected region; *N* = the total number of pixels of that color.

#### 2.4.6 Global Moran’s I.

Global Moran’s I is used to describe the statistical measure of spatial autocorrelation for the entire study area and the spatial dispersion of experimental data. We used this method in the experiment to verify the spatial correlation of various natural landscape indicators, rejecting the null hypothesis in spatial statistics. Typically, a statistical significance test of Moran’s I is conducted to determine whether the correlation is coincidental or if the entire study area indeed exhibits spatial autocorrelation. The principle is presented in Formula 3 [75]:


$$I=\frac{n\sum_{i=1}^{n}\sum_{j=1}^{n}w_{ij}\left(x_{i}-\overset{―}{x}\mathrm{\backslash rightleft}(x_{j}-\overset{―}{x}\right)}{\sum_{i=1}^{n}\sum_{j=1}^{n}w_{ij}\sum_{i=1}^{n}{\left(x_{i}-\overset{―}{x}\right)}^{2}}$$ (3)

*M* represents the number of spatial units, xi is the observed value of the spatial unit, $\overset{―}{x}$ is the mean value of the observations across spatial units, and wij represents the elements of the spatial weight matrix 𝑊. Global Moran’s I determines whether there is a positive or negative correlation across the area. The theoretical range of 𝐼 is from -1–1. An 𝐼 > 0 indicates spatial positive autocorrelation, suggesting that spatial units with high (or low) observations are surrounded by other units with high (or low) observations. An 𝐼 < 0 means the data exhibits spatial negative autocorrelation, indicating that units with high (or low) observations are clustered together with those having low (or high) observations. An 𝐼 = 0 implies no spatial autocorrelation, suggesting a random spatial distribution of observations.

#### 2.4.7 Getis-Ord Gi*.

The Getis-Ord Gi* statistic [76] aims to calculate the statistical significance of areas with the highest positive or negative emotions. It can also analyze different natural landscape indicators’ spatial clustering distribution characteristics. Hotspot analysis maps are commonly used to display the geographical distribution characteristics of data and assist in identifying spatial patterns such as clustered “hotspots” or dispersed “cold spots.” Using the Gi* statistic, after validating spatial autocorrelation, we further displayed hotspots and cold spots of emotion scores in UGC texts on the map. It can be calculated using Formula 4:


$$G_{i}=\frac{\sum_{j=1}^{n}\omega_{i,j}x_{j}-\overset{―}{x}\sum_{j=1}^{n}\omega_{i,j}}{s\sqrt{n/(n-1sum_{j=1}^{n}\omega_{i,j}^{2}-1/(n-1){\left(\sum_{j=1}^{n}\omega_{i,j}\right)}^{2}}}$$ (4)

In the formula, x_j_ represents the count or emotional value of *j*, the term *w*_*ij*_ defines the spatial weight between events *i* and *j*, and n is the total number of events. $\overset{―}{x}$ and *S* are the sample mean and standard deviation of *x*_*j*_, respectively. The generated z-scores and p-values can indicate the locations where elements with high or low values are spatially clustered. For statistically significant z-scores, the larger the z-score, the tighter the clustering of high values (hotspots). Conversely, for statistically significant negative z-scores, the smaller the z-score, the tighter the clustering of low values (cold spots).

#### 2.4.8 EXtreme Gradient Boosting (XGBoost) model and SHapley Additive exPlanation (SHAP) analysis.

To analyze and compare the correlations between various natural landscape indicators and tourists’ emotions in high-temperature and non-high-temperature conditions, we utilized the XGBoost algorithm to build a regression model and employed the SHAP method for interpretability analysis. XGBoost excels at managing complex nonlinear relationships and offers exceptional parallel processing capabilities, effectively mitigating potential overfitting issues in machine learning regression models. The SHAP method elucidates how different indicators influence the model’s output by calculating, comparing, and contrasting marginal contributions within the model. Furthermore, it incorporates easily interpretable elements such as feature importance analysis, waterfall plots, and interaction value analysis, accurately evaluating and visualizing the contribution of each research indicator. The model effectively captures the patterns of tourists’ sentiment perception, demonstrating robust performance in both temperature conditions, which provides a solid foundation for subsequent experiments and applications. The principle is shown in Formula 5:


$$g(z^{\prime })=\emptyset_{0}+\sum_{i=1}^{M}\emptyset_{i}{z^{\prime }}_{i}$$ (5)

$g(z^{\prime })$ represents the predicted value of the sentiment index impact of landscape element $z^{\prime }$, $\emptyset_{0}$ represents the average indicator of landscape element composition, *M* represents the number of variables in the model, and $\emptyset_{i}$ represents the SHAP value of the ith study indicator.

## 3. Results

### 3.1. Sample granularity statistics

**Fig 3:**
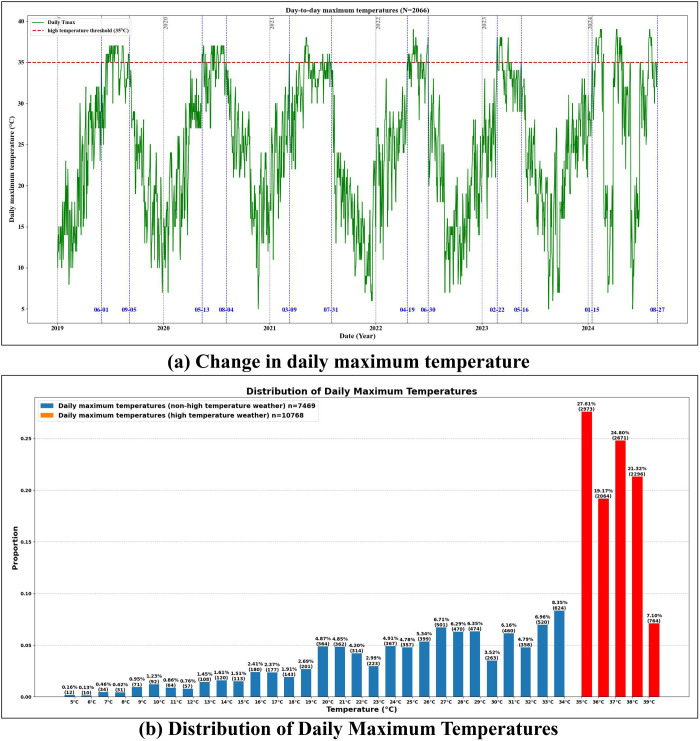
Distribution of Daily Maximum Temperatures.

To better illustrate how high-temperature weather affects tourists’ perception and emotional experience of natural landscapes, Fig 3 presents the variation in Wuyishan City’s daily maximum temperatures and the corresponding data granularity distribution. As shown in Fig 3(a), among 2,066 observation days (n = 2,066) from 2019 to 2024, the daily maximum temperature exhibits obvious seasonal fluctuations, peaking around mid-year each year. The blue dashed lines on the y-axis represent the earliest appearance and latest end of high-temperature weather each year, while the red dashed line on the x-axis marks 35°C as the threshold for defining high-temperature weather. Although this threshold is frequently exceeded during summer, it largely follows an unstable high-temperature trend—for instance, in 2023 and 2024, high temperatures first appeared in spring. These patterns confirm that, in recent years, Wuyishan City in Fujian Province has experienced increased high-temperature events. Fig 3(b) shows the granularity distribution of UGC (User-Generated Content) data by daily maximum temperature, divided into non-high-temperature days (<35°C) and high-temperature days (≥35°C). Out of the 2,066 observation days, there were 7,469 non-high-temperature records (n = 7,469) and 10,768 high-temperature records (n = 10,768). This indicates a prominent high-temperature phenomenon in Wuyishan City and suggests that tourists are more inclined to travel during high-temperature periods. Notably, daily maximum temperatures of 35°C (27.61%), 37°C (24.80%), and 38°C (21.32%) yielded the greatest volume of UGC data, underscoring the concentration of ecotourism during high-temperature weather. This trend further demonstrates that visitors to natural ecotourism destinations are frequently exposed to higher-temperature environments.

**Fig 4:**
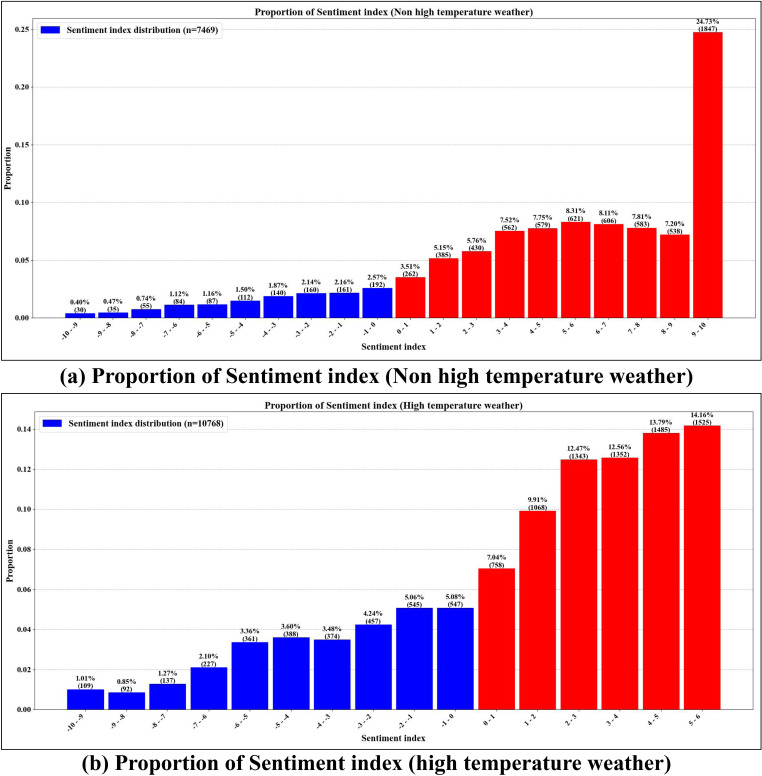
Proportion of Sentiment index.

Fig 4 illustrates the distribution of tourists’ sentiment indices under non-high and high-temperature conditions. The results indicate that negative emotions account for 30.1% under high-temperature conditions, while positive emotions make up 69.9%. In contrast, negative emotions are significantly lower at 14.1% under non high temperature conditions, with positive emotions reaching 85.9%. Additionally, in non-high-temperature weather (<35°C), tourist sentiment indices are predominantly positive, with a strong concentration in the 9–10 score range, accounting for 24.73% (n = 1847) of the data. In comparison, overall sentiment scores are lower under high-temperature conditions, peaking in the 5–6 score range, with a share of 14.16% (n = 1525), which is noticeably lower than in non high temperature conditions. This suggests that high-temperature weather contributes to increased negative emotions among tourists, potentially impacting their overall travel experience.

### 3.2. Results of global moran’s I, z-score, and p-value

Using Global Moran’s I analysis in ArcMap software, we conducted spatial autocorrelation validation of natural landscape indicators under high temperature (HT) and non high temperature (NH) conditions. Table 3, titled “Results of Global Moran’s I, Z-Score, and P-Value,” presents the calculation results for different indicators, where “HT” represents high-temperature weather and “NH” represents non-high-temperature weather. It can be observed that most indicators exhibit significant spatial autocorrelation with clustering tendencies, with Moran’s I values ranging from 0.001 to 0.076. Additionally, most indicators have Z-scores greater than 2.58, surpassing the threshold for statistical significance, indicating highly significant spatial clustering patterns for the different research indicators. All indicators have P-values less than 0.05, suggesting that the observed spatial distribution patterns are improbable to have occurred by chance.

**Table 3:** Results of Global Moran’s I, z-score, and p-value.

Research indicators	Moran’s I (HT)	Z-score (HT)	P-value (HT)	Moran’s I (NH)	Z-score (NH)	P-value (NH)
**Sentiment index**	-0.005	-5.628	<0.001	0.013	68.326	<0.001
**Openness**	0.002	8.770	<0.001	0.005	26.928	<0.001
**Aquatic rate**	0.076	291.411	<0.001	0.027	144.998	<0.001
**Greenness**	0.001	7.239	<0.001	0.006	33.792	<0.001
**Paving degree**	0.008	3.767	<0.001	0.005	3.729	<0.001
**Ground exposure**	0.002	2.314	<0.050	0.004	23.803	<0.001
**Enclosure**	0.010	40.394	<0.001	0.006	32.033	<0.001
**Color complexity**	0.001	7.898	<0.001	0.006	4.173	<0.001
**Visual Entropy**	0.002	2.313	<0.050	0.007	4.475	<0.001

The results indicate that the Sentiment Index shows significant differences in spatial clustering characteristics between high-temperature and non-high-temperature conditions. Under high-temperature conditions, the Moran’s I for the Sentiment Index is -0.005 with a Z-score of -5.628, whereas for non-high-temperature conditions, Moran’s I is 0.013 with a Z-score of 68.326. This indicates that tourists’ emotions display significant spatial dispersion under high-temperature conditions, while the opposite is true under non-high-temperature conditions. The Aquatic Rate indicator also reveals differences in spatial clustering under varying conditions. In high-temperature conditions, the Z-score of the Aquatic Rate is 291.411, while in non-high-temperature conditions, it is 144.998, suggesting that the spatial clustering characteristics of water elements favored by tourists are more pronounced during high temperatures. Conversely, the Openness indicator demonstrates a more clustered spatial distribution under non-high-temperature conditions.

### 3.3. Hotspot analysis results

After validating global spatial autocorrelation using Global Moran’s I, this section presents further hotspot analysis results among different indicators. We utilized Gi-Bin values from the Getis-Ord Gi* statistic to represent cold spots and hotspots of various indicators in space. Gi-Bin values ranging from 1 to 3 indicate hotspot areas, with warmer colors (red) representing more concentrated hotspots. Conversely, Gi-Bin values from -1 to -3 reflect cold spot distributions, with cooler colors (blue) indicating more clustered cold spots. Areas with a Gi-Bin value of 0 represent regions where a particular indicator’s tendency is insignificant or where the numerical distribution is relatively uniform. Figure 3, titled “Hotspot Analysis Results of Sentiment Index,” illustrates the distribution characteristics of Gi-Bin values corresponding to tourists’ sentiments, using different confidence levels: Gi-Bin values of 1–3 and -1 to -3 correspond to confidence levels of 90%, 95%, and 99%, respectively.

#### 3.3.1. Hotspot analysis results of sentiment index.

**Fig 5:**
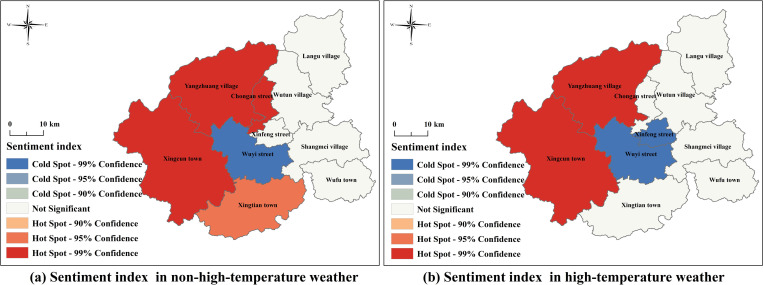
Hotspot analysis results of the Sentiment index Note: Based on the standard map production of the National Natural Resources Department’s Standard Map Service website GS(2019) 1822, the base map boundaries have not been modified. (http://bzdt.ch.mnr.gov.cn/download.html?searchText=GS(2019)1822).

In Fig 5, red areas (Hot Spot - 99% Confidence) indicate regions with a relatively positive emotional tendency, while blue areas (Cold Spot - 99% Confidence) represent regions with a pronounced negative emotional tendency. It is evident that regardless of weather conditions, most negative emotions in cold spots among tourists are concentrated in the Wuyi Street area in the center of Wuyishan City. Comparatively, the spatial distribution of sentiment cold and hot spots under non-high-temperature conditions shows a more uniform transition between sub-cold spots (Cold Spot - 95%/90% Confidence) and sub-hot spots (Hot Spot - 95%/90% Confidence). This indicates that under high-temperature conditions, tourists’ emotional expressions exhibit more extreme disparities, with a higher prevalence of low-scoring negative sentiment texts or more significant differences in sentiment index scores, widening the gap between cold and hotspot regions.

#### 3.3.2. Hotspot analysis results of independent variable.

**Fig 6:**
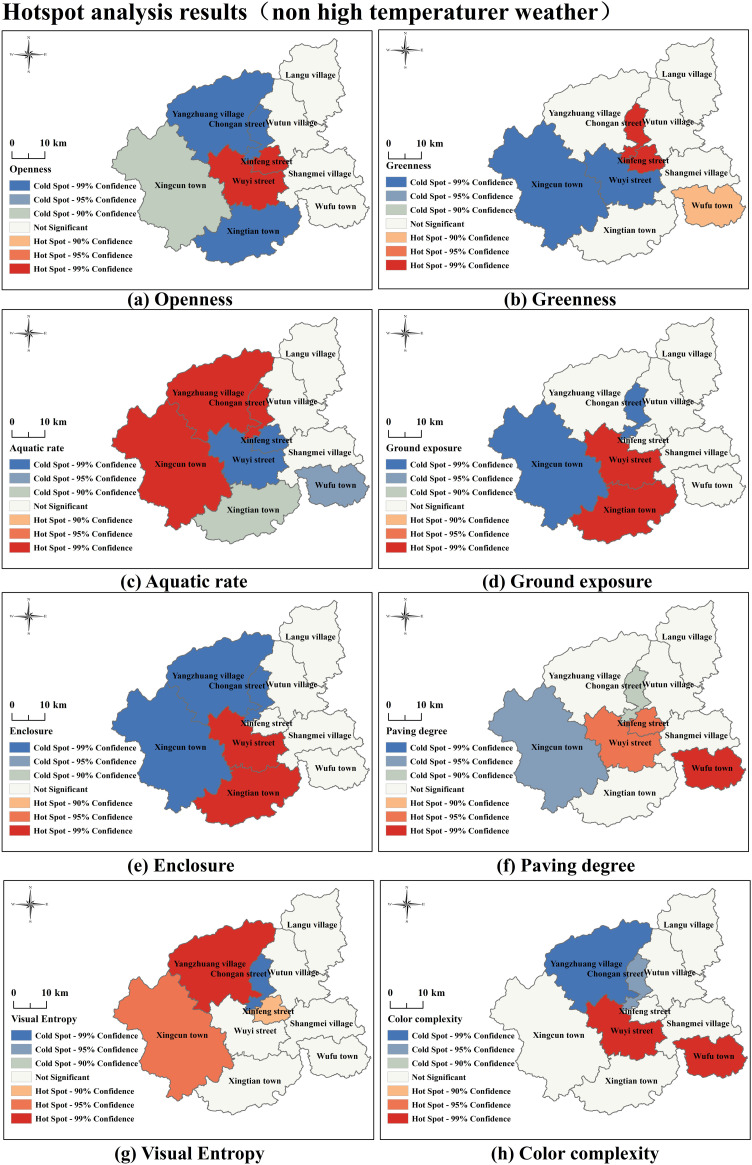
Hotspot analysis results (non high temperaturer weather) Note: Based on the standard map production of the National Natural Resources Department’s Standard Map Service website GS(2019) 1822, the base map boundaries have not been modified. (http://bzdt.ch.mnr.gov.cn/download.html?searchText=GS(2019)1822).

**Fig 7:**
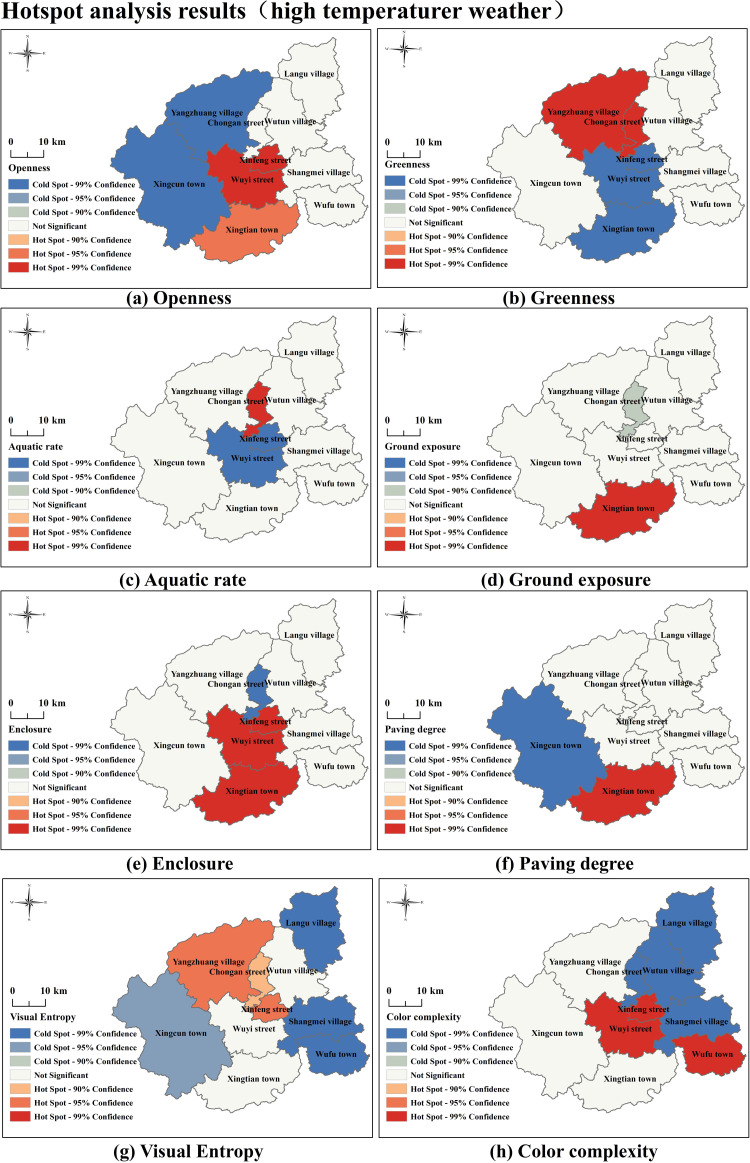
Hotspot analysis results (high temperaturer weather) Note: Based on the standard map production of the National Natural Resources Department’s Standard Map Service website GS(2019) 1822, the base map boundaries have not been modified. (http://bzdt.ch.mnr.gov.cn/download.html?searchText=GS(2019)1822).

Fig 6 and 7 present the hotspot analysis results of independent variables under different temperature conditions, including Landscape Elements and Visual Quality. We observe that under non-high-temperature conditions, most indicators show significant cold or hot spots spread outward from the central area, with uniform transitions between cold and hotspots. In contrast, under high-temperature conditions, indicators exhibit stronger clustering effects of cold and hotspots, especially with greater disparities in hotspot clustering characteristics compared to non-high-temperature conditions—for instance, the indicators Greenness, Ground Exposure, and Paving Degree. This suggests that high-temperature weather significantly affects tourists’ perception patterns of landscape elements, leading to enhanced or diminished attractiveness of certain landscape features, resulting in more concentrated emotional and behavioral responses. High temperatures reduce the hotspot areas of Aquatic Rate and Visual Entropy. Under varying temperature conditions, tourists’ perception effects of Color Complexity and the spatial clustering characteristics of cold and hotspots are relatively similar.

To explore the interaction relationships among different indicators and how they influence tourists’ emotions, we present SHAP interpretability analysis results in the following section to further investigate the complex relationships among various variables.

### 3.4. SHAP feature importance and beeswarm plot analysis

**Fig 8:**
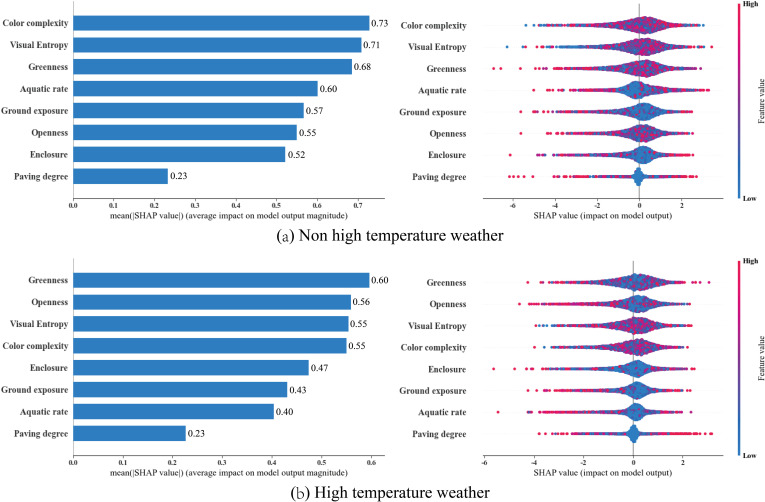
SHAP feature importance.

This section further explores the impact of different natural landscape indicators on tourist emotions through SHAP feature importance, heatmap analysis, and waterfall analysis. The left side of Fig 8 displays the SHAP feature importance, where each feature’s global importance is considered as the mean absolute SHAP value across all samples, sorted by their contribution. The right side shows the SHAP Beeswarm plot, providing a dense summary of how the dataset’s indicator values influence the model’s output.

Fig 8(a) and 8(b) illustrate the contributions of different natural landscape perception indicators to tourists’ emotions under non-high-temperature and high-temperature conditions, respectively. We can clearly compare the analytical results under different weather scenarios. The findings are as follows:

(1) Non High Temperature Conditions: The four indicators with the most significant impact on tourists’ emotions are Color Complexity (0.73), Visual Entropy (0.71), Greenness (0.68), and Aquatic Rate (0.60). Their importance scores are all greater than or equal to 0.6, substantially surpassing the contribution scores of the remaining four indicators: Ground Exposure (0.57), Openness (0.55), Enclosure (0.52), and Paving Degree (0.23). Notably, from the SHAP Beeswarm plot on the right, we observe that the contribution values of Aquatic Rate are mostly less than zero, contrasting with the contributions of other indicators. This indicates that under non-high-temperature conditions, Aquatic Rate does not significantly contribute to positive emotions. Moreover, most indicators such as Color Complexity, Visual Entropy, and Greenness generally have positive contributions to the model.
(2) High Temperature Conditions: In contrast, under high-temperature conditions, the four indicators with the most significant impact are Greenness (0.60), Openness (0.56), Visual Entropy (0.55), and Color Complexity (0.55). Their importance scores are all greater than or equal to 0.55. It is noteworthy that high-temperature conditions overall weaken the effect of natural landscape perception on emotions, making Greenness and Openness the most critical natural landscape perception elements in this scenario. Although high temperatures significantly reduce the importance of the Aquatic Rate indicator, under these conditions, Aquatic Rate can produce more positive effects. This suggests that water landscapes still hold unique value during high temperatures, while Greenness and Openness play more vital roles in alleviating discomfort caused by heat and enhancing overall environmental appeal.

In this subsection, we utilized SHAP feature importance and SHAP Beeswarm plots to observe the contributions and importance of natural landscape elements to tourists’ emotions under different conditions. To further explore their nonlinear relationships and interactions, we will integrate SHAP Partial Dependence Plots, SHAP Waterfall Plots, and SHAP Force Plot analyses in the subsequent sections.

### 3.5. Boxplot and SHAP Partial Dependence Analysis

**Fig 9:**
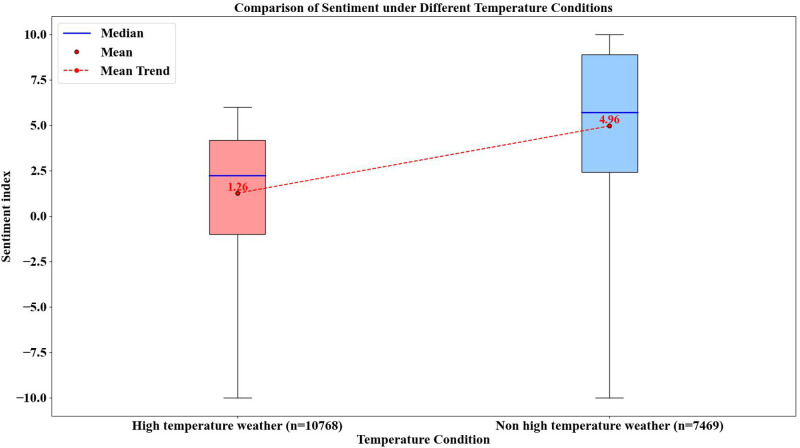
Boxplot of Sentiment under Different Temperature Conditions.

Fig 9 presents a boxplot comparison of tourists’ sentiment indices under different temperature conditions. The blue line in the boxplots represents the median, while the red dots indicate the mean values. The red dashed lines connecting different boxplots illustrate the trend of mean value changes. The figure clearly shows that under non-high-temperature conditions (<35°C), both the median and mean sentiment indices are significantly higher than those under high-temperature conditions (≥35°C). Specifically, the mean sentiment index in non-high-temperature weather is 4.96, with a relatively high median, reflecting a more positive emotional tendency. In contrast, under high-temperature conditions, the mean sentiment index drops to just 1.26, with a notably lower median, indicating a shift toward a more neutral emotional state.

**Fig 10:**
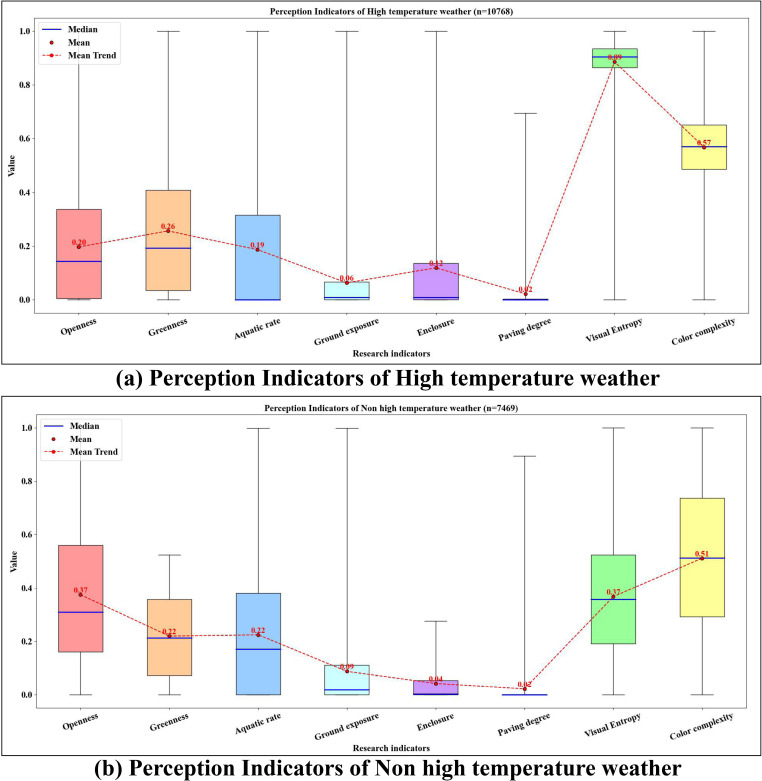
Boxplot of perception indicators.

Fig 10 further analyzes the differences in tourists’ perceptions of natural landscape elements under varying temperature conditions. Under high-temperature conditions, the perception of Visual Entropy is notably enhanced, with an average value of 0.89, significantly higher than all other perception indicators. In contrast, Paving Degree (0.02) and Ground Exposure (0.06) are perceived at much lower levels. Comparatively, in non-high-temperature conditions, tourists’ perception of Greenness (0.22), Color Complexity (0.51), and Visual Entropy (0.72) is slightly weaker, while Openness (0.37) shows a significant increase in preference. The perception levels of Paving Degree (0.02) and Enclosure (0.04) remain relatively low. These results highlight the substantial impact of rising temperatures on tourists’ preferences for natural landscape elements, particularly in terms of visual quality perception.

**Fig 11:**
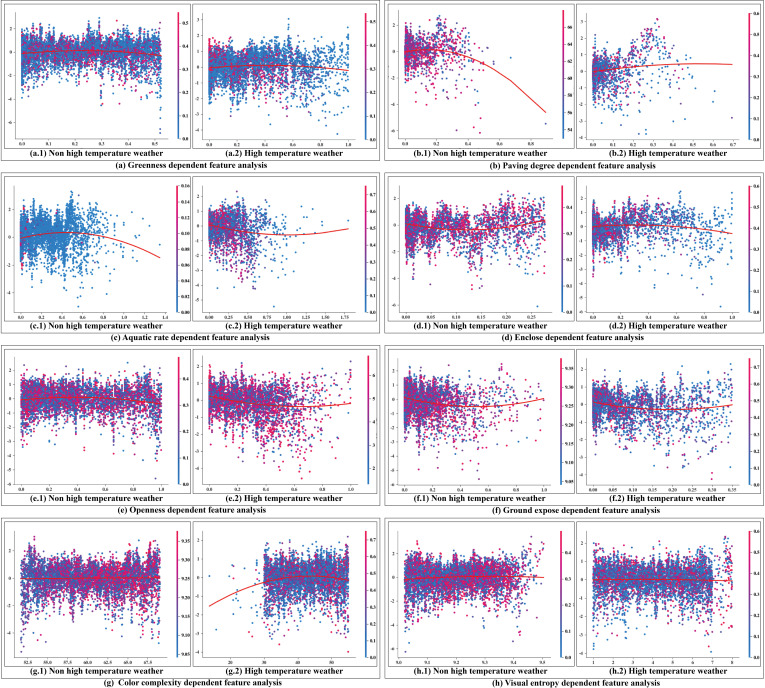
SHAP Partial Dependence Plots analysis.

Fig 11 presents the SHAP Partial Dependence Plots, displaying the marginal effects of each natural landscape indicator on the tourists’ sentiment index within the model. Fitted curves are included to better compare the nonlinear associations between indicators under different weather conditions. The results are as follows:

(1) The three indicators—Greenness, Ground Exposure, and Visual Entropy—show relatively stable contribution values under different weather conditions. This indicates that these landscape elements have a consistent and stable impact on tourists’ emotions regardless of temperature variations. Notably, the contribution threshold of Greenness reaches higher levels under high-temperature conditions, approaching 3 in Fig 11(a), whereas it only reaches 2 in Fig 11(b) and even shows stronger negative contributions in some cases. This further demonstrates that Greenness has a significant moderating effect in high-temperature environments, effectively mitigating adverse impacts on tourists through visual comfort. In milder climates, the effect of Greenness is relatively unstable due to the influence of other landscape elements.
(2) The indicators Paving Degree, Aquatic Rate, and Color Complexity exhibit substantial nonlinear differences in their contributions to tourists’ emotions under varying weather conditions. Under non-high-temperature conditions, the contribution of Paving Degree significantly decreases after exceeding 0.4, whereas under high-temperature conditions, its contribution remains relatively stable and shows a gradual upward trend. For Aquatic Rate, after reaching a peak at 0.6 under non-high-temperature conditions, its contribution declines, and most values indicate a negative effect. In contrast, under high-temperature conditions, Aquatic Rate shows a slow decrease before reaching approximately 0.75, after which it gradually increases. Color Complexity demonstrates a stronger upward trend under high-temperature conditions; as the Color Complexity increases, its contribution to tourists’ emotions also rises steadily.
(3) Although the contribution distribution of the Openness indicator is more unstable under high-temperature conditions, it exhibits significant differences in framing preferences across different weather scenarios. Under non-high-temperature conditions, the Openness values in tourists’ photos are relatively uniformly distributed between 0 and 1. In contrast, under high-temperature conditions, most Openness values range between 0 and 0.6. This difference further indicates that Openness has a significantly altered appeal to tourists under varying temperatures. In non-high-temperature settings, open landscapes enhance tourist satisfaction, reflecting a higher visual preference. However, in high-temperature environments, tourists’ preference for open landscapes diminishes, possibly due to the lack of shade and cooling functions.

In this section, we performed Partial Dependence Plots Analysis on the important indicators identified in Section 3.3. Many indicators exhibited complex nonlinear effects and coupling relationships. Therefore, in the next section, we will sample the indicators and conduct SHAP Waterfall Plot and Force Plot analyses to further clarify how different indicators influence tourists’ emotions.

### 3.6. SHAP waterfall and force plot Analysis

In our study, we initially discovered some nonlinear associations among indicator distributions. Consequently, we employed SHAP Waterfall Plot and Force Plot analyses to further explore the marginal effects of various variables on tourists’ emotions. The waterfall plot is designed to present explanations for individual predictions by taking a single instance as input. It starts from the expected value of the model’s output, with each subsequent row indicating whether each feature contributes positively (red) or negatively (blue), illustrating how values are altered from the model’s expected output to the predicted output. The SHAP Force Plot offers explanations for multiple samples.

**Fig 12:**
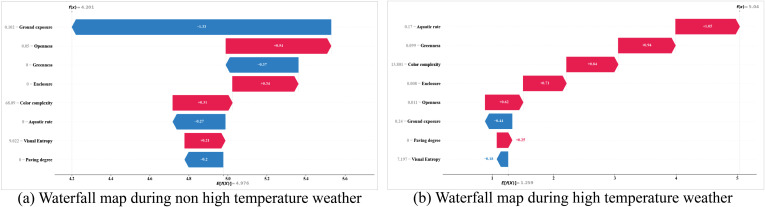
SHAP waterfall analysis.

**Fig 13:**
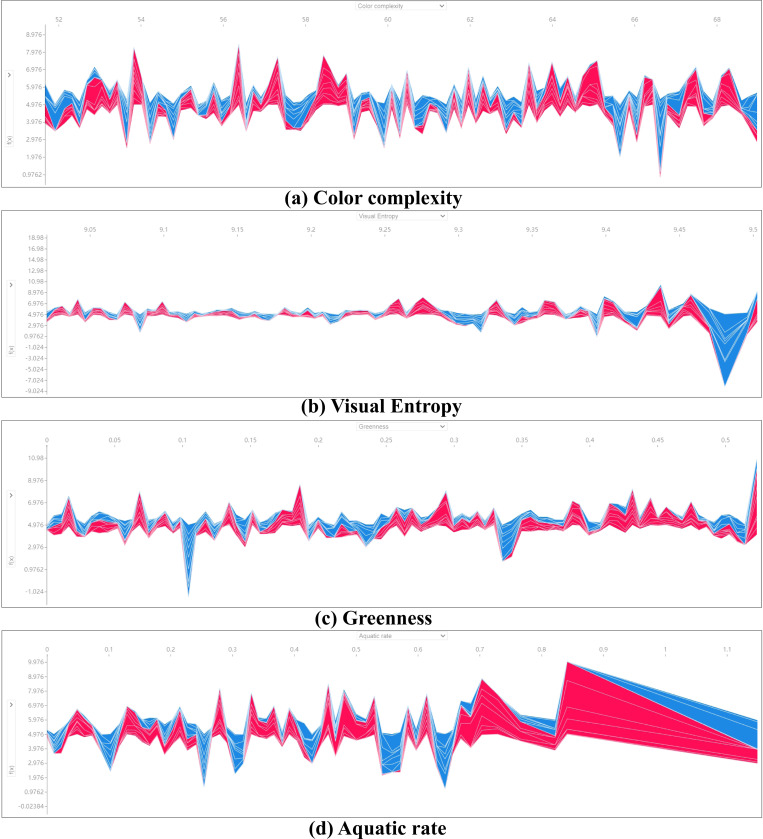
SHAP Force plot analysis of non high temperature weather.

**Fig 14:**
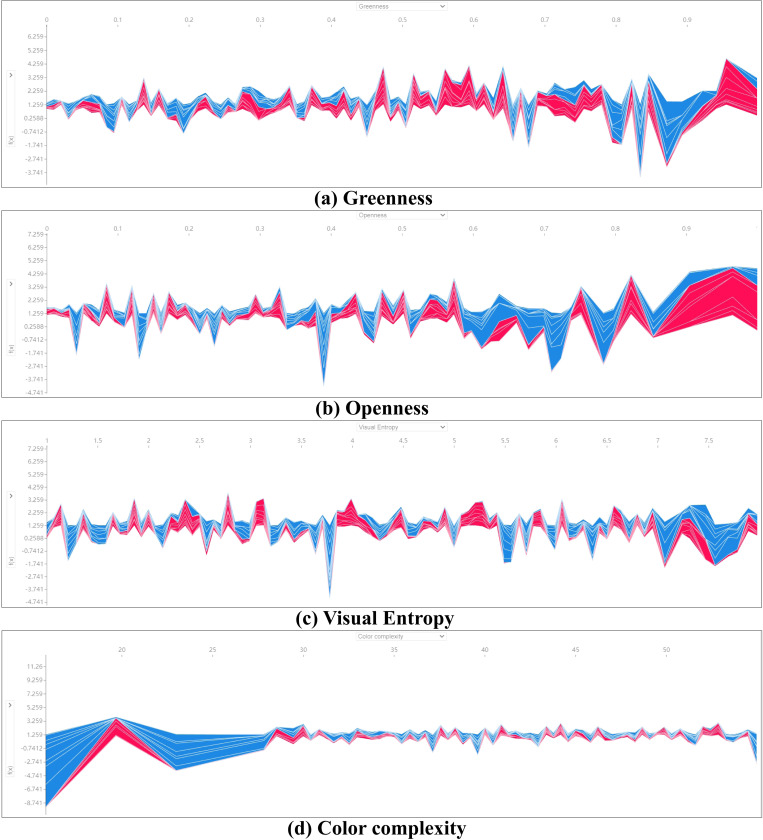
SHAP Force plot analysis of high temperature weather.

(1) Fig 12, titled “SHAP Waterfall Analysis,” compares the contribution results of sampled indicators for single predictions under different weather conditions, revealing significant differences between high-temperature and non-high-temperature scenarios. Under non-high-temperature conditions, indicators with significant negative contributions are Ground Exposure (-1.33), Greenness (-0.37), and Aquatic Rate (-0.27), while those with significant positive contributions are Openness (0.54), Enclosure (0.34), and Color Complexity (0.31). Under high-temperature conditions, apart from Ground Exposure (-0.44) and Visual Entropy (-0.18) negatively impacting tourists’ emotions, most other indicators like Aquatic Rate (1.05), Greenness (0.94), and Color Complexity (0.84) show stable positive contributions. Additionally, appropriate artificial environmental elements can generate positive effects under high-temperature conditions, such as Enclosure (0.71) and Paving Degree (0.25).
(2) The results demonstrate significant disparities in the contribution values of the Aquatic Rate indicator under different conditions. This suggests that water landscapes have a notable emotional regulation effect in high-temperature environments (contribution of 1.05), effectively enhancing tourists’ overall experience. Moreover, in extreme climatic conditions, landscape design should focus on the functional differences of various landscape elements to better meet tourists’ needs. For instance, indicators like Greenness and Openness also uniquely contribute to alleviating the negative emotional impacts of high temperatures. It is important to note that Visual Entropy shows negative contributions under high-temperature conditions, indicating that complex visual information may increase cognitive load and deteriorate emotional experiences in extreme heat. Therefore, reducing visual complexity might help improve comfort and emotional stability for tourists during high temperatures.
(3) In Fig 13 and 14, to further explain multiple samples and explore the coupling patterns of different important indicators under varying conditions, we conducted a Force Plot Analysis on the top four indicators with the highest overall impact on the model from the SHAP feature importance. In Fig 9, it is evident that under non-high-temperature conditions, the contributions of Color Complexity and Aquatic Rate fluctuate more significantly compared to Visual Entropy and Greenness, whose impacts on emotions are more stable. This further explains why the positive effects of Aquatic Rate are not significant under non-high-temperature conditions. Conversely, under high-temperature conditions, the overall benefit trends of important indicators are more stable, especially the more pronounced roles of Greenness and Openness. This indicates that during high temperatures, tourists are more inclined to favor landscape elements with cooling effects and openness characteristics, thereby alleviating discomfort caused by heat.

## 4. Discussion

### 4.1. New insights into emotional perception of natural landscapes

This study provides evidence on how landscape perception under different weather conditions affects tourists’ emotions and further explores their coupling effects from the perspectives of geographic analysis and machine learning modeling. Although Wuyishan City boasts numerous ecotourism attractions, spatial autocorrelation results indicate that various landscape indicators exhibit highly clustered spatial patterns. We speculate that this is due to the distribution characteristics of natural landscape resources in Wuyishan, leading to significant spatial dependence and clustering in ecotourism attractions and landscape distributions, similar to the findings of Peng et al. [77]. Notably, tourists’ emotional perceptions differ significantly under different weather conditions, aligning with the experimental results of Yan et al. [78]. Specifically, under high-temperature conditions, tourists’ emotions display obvious spatial dispersion, possibly due to increased distracted walking behavior induced by the perception of heat [79]. This results in tourists’ photography preferences not exhibiting a clustering trend. The significant differences in the hot and cold spot distributions of Greenness, Ground Exposure, and Paving Degree under different weather conditions further validate this viewpoint. Although our experimental results found that the spatial clustering characteristics of aquatic elements preferred by tourists are more pronounced under high-temperature conditions, the Aquatic Rate does not significantly contribute to positive emotions under non-high-temperature conditions. This contrasts sharply with previous studies demonstrating that water elements significantly enhance people’s emotions and vitality [80,81]. One possible explanation is that the landscape configurations of ecotourism attractions have mostly not undergone large-scale planning and design and are somewhat related to ecological landscape pattern characteristics [82]. Therefore, in natural landscape environments rich in water bodies, suitable green indices, and diverse plant community structures, tourists’ overall perception experience is already positive, leading to the unique positive effects of waterscapes being less significant in this context. This phenomenon is further explained by the viewpoints of Li et al. [83] and the high proportion of positive emotions among tourists observed in our study.

Additionally, there may be various conflicts between landscape protection, water management, ecological governance, and societal interests in natural landscapes [84], which can influence the changes in tourists’ emotions observed in the experimental results. Our findings also indicate that green plant indicators under high-temperature conditions play a more crucial role in alleviating discomfort caused by heat and enhancing the overall environmental appeal, corroborating previous studies suggesting that natural vegetation can reduce stress and promote recovery [85]. It is noteworthy that the overall benefit trend of key indicators under high-temperature conditions is more stable, whereas under non-high-temperature conditions, the trends are more variable, which differs from the findings of Yan et al. [86]. Generally, tourists exhibit more stable perception levels in comfortable temperatures. This discrepancy may be because climate affects tourists’ travel processes [87]. Although high temperatures impact tourists’ emotional expressions, they still tend to prioritize their preferred natural landscape destinations, resulting in more stable quantified landscape perception levels in UGC data [88]. Furthermore, the higher visual complexity of natural landscapes also significantly impacts tourists. Although Color Complexity and Visual Entropy are mostly at high values under high-temperature conditions, our findings differ from previous research suggesting that landscapes with higher variation are often associated with lower visual quality [89,90]. In our experiment, the visual quality indicators generally showed important and stable trends. This may be because, under high-temperature conditions, rich colors and higher visual complexity can distract tourists from the discomfort of heat, enhancing environmental pleasantness. Previous studies have also demonstrated that as the Universal Thermal Climate Index increases, the influence weight of aesthetic comfort on overall comfort rises by 85.4% [91], indirectly indicating the importance of visual elements on tourists’ overall comfort under high-temperature conditions.

Moreover, the potential negative effects on tourists’ emotions when different variables interact and weather conditions change further highlight the uncertainty of tourists’ perceptions in complex landscapes [92]. The increase in Openness consistently shows a stable positive effect under different weather conditions, consistent with the findings of Tabatabaie et al. [93]. This may be because visually open landscapes meet people’s aesthetic needs for natural environments, promoting social interaction and the generation of positive emotions. In actual natural landscape planning and conservation, we should fully consider how to organize and optimize ecotourism attractions as a whole, treating accessible natural landscapes as an important component in enhancing tourists’ emotions. Therefore, in the following sections, we propose sustainable optimization strategies for ecotourism and natural landscape conservation based on the issues and results identified in the research, particularly in constructing response strategies for ecotourism attractions under sustained high-temperature conditions. We also explore how to leverage the potential advantages of natural landscapes to promote tourists’ emotions, enabling planners and practitioners to apply our experimental data results and methods.

### 4.2 Recommendations for the sustainable development of natural landscapes in ecotourism destinations

#### 4.2.1 Perform real-time environmental assessment and strategy adjustment.

Our experiment shows that in high-temperature conditions, the proportion of tourists experiencing negative emotions significantly increases and is likely influenced by elements of the natural landscape. Implementing real-time environmental assessments and adjusting strategies is essential for improving tourist experiences, reducing negative emotions caused by high temperatures, and achieving the goals of both landscape systems and social-ecological systems. [94]. Before this, it is necessary to build corresponding databases and datasets for specific areas and issues requiring optimization. Scenic area managers should establish comprehensive environmental monitoring systems, collecting data on temperature, humidity, tourist density, and other factors in real time to promptly grasp microclimate changes within the scenic area. Moreover, machine learning methods can be utilized to quantify and analyze natural landscapes and tourist reviews in ecotourism attractions, enabling the customization of real-time management plans. Attention should also be paid to the impact of climate change and tourist flows on landscape perception [95]. Technical means can be employed to monitor review information and landscape conditions in real time, thereby improving management efficiency in addressing related issues, especially concerning natural landscape maintenance. Additionally, while low or mild temperatures typically do not cause extreme heat stress, destination managers can leverage real-time monitoring systems (e.g., temperature, humidity, visitor density) to identify potential environmental or crowd-related issues in advance. This proactive approach allows for targeted landscape maintenance and visitor flow management before peak seasons, ensuring sustained comfort and accessibility. Such active management not only prevents declines in visitor experience due to overlooked microclimate changes but also enhances preparedness for unexpected challenges during high-temperature periods.

#### 4.2.2 Enhancing natural landscape infrastructure in ecotourism destinations.

The experimental results indicate that under high-temperature conditions, tourists’ emotions are more significantly influenced by specific natural landscape elements, particularly Greenness, Openness, Visual Entropy, and Color Complexity. This suggests that tourists in ecotourism not only prioritize the richness of vegetation but also seek diverse and layered landscape experiences. Therefore, enhancing natural landscape infrastructure and creating rich and varied landscape features are crucial for elevating tourists’ positive emotions. Our findings show that thoughtful artificial environmental constructions can enhance tourists’ positive emotions to a certain extent. To ensure that tourists gain emotional value and support the sustainable development of ecotourism under varying temperature conditions, it’s essential to strike a balance between natural landscape visual elements and artificial environments. While maintaining ecological balance, the construction of landscape infrastructure within forest tourism areas should be reinforced, including the installation of minimally invasive wooden walkways, observation stations, and secluded spots. These facilities allow visitors to engage with nature closely without harming the environment and enhance tourists’ comfort when visiting specific attractions under high-temperature conditions. Supplement cooling and comfort facilities based on the quantitative analysis of water bodies, greenery, and visual elements. In identified hotspot areas, install mobile cooling stations, shaded corridors, or convenient water access points to mitigate the negative emotional impact of high temperatures. These measures ensure a more comfortable and enjoyable experience, reducing thermal stress while enhancing visitor satisfaction and engagement with the natural environment. Additionally, appropriate restoration or promotion of natural ecosystems can stimulate people’s imagination and emotional resonance [96], enhancing the sustainability of the local ecotourism economy. Finally, in developing ecotourism points of interest, scenic areas should adjust strategies based on tourists’ actual experiences and weather conditions, such as limiting visitor numbers, providing online educational resources, or designing equipment that enhances tourists’ comfort under high-temperature conditions. This helps balance the pressure on natural landscapes and tourists’ needs [97]. It is worth noting that enhancing natural landscape infrastructure is more efficiently implemented during non-high-temperature periods and provides long-term benefits to visitor experiences. For example, upgrading trails, observation decks, and vegetation landscapes during mild seasons ensures that infrastructure improvements are completed when visitor flow is stable, minimizing disruptions during peak heat periods. Additionally, increasing green coverage and optimizing water landscapes during non-high-temperature seasons helps create a year-round enjoyable environment, while offering significant cooling and emotional regulation effects during extreme heat. Thoughtfully designed blue-green spaces and diverse color compositions further enhance visitor relaxation and enjoyment. By improving natural landscape infrastructure in ecotourism destinations, both cooling and emotional benefits can be achieved in extreme heat conditions while maintaining high scenic appeal throughout all seasons.

#### 4.2.3 Enhancing landscape connectivity and seasonal adaptation management strategies.

Our study shows that high-temperature conditions amplify the emotional impact of natural landscape elements, and the spatial distribution and changes of these elements are crucial to the tourist experience. To improve tourist satisfaction and promote the sustainable development of ecotourism under high-temperature conditions, strengthening landscape connectivity and implementing seasonal adaptation management strategies are particularly important. First, constructing continuity between landscapes is vital for the sustainability of ecosystems [98], and enhancing the value of landscape connectivity does not conflict with natural landscape conservation [99]. Based on our experimental results, in practice, continuous green spaces and shaded walkways can be planned to connect various scenic spots and rest areas, forming an integrated landscape network. Integrating high-temperature adaptation measures—such as shaded corridors and water connectivity—with routine ecological maintenance can create a comprehensive network of greenways, water systems, and functional nodes. This approach not only enhances visitor walking experiences during high-temperature periods but also adds ecological and recreational value throughout the year. Encouraging local community participation through co-management mechanisms can further optimize this strategy. By involving residents and local businesses in landscape enhancement, plant selection, and maintenance, the initiative can improve landscape continuity, conservation efficiency, and service quality. Such collaborative efforts ensure that the study’s recommendations are implemented sustainably, fostering long-term resilience and adaptability in both ecological and tourism management. This not only provides tourists with continuous shade and cooling effects but also enhances the integrity and aesthetics of the landscape. Additionally, increasing the connectivity between water bodies—such as combining streams, ponds, and artificial waterscapes—leverages the cooling and beautifying functions of water bodies, enhancing tourists’ comfort and emotional experience under high-temperature conditions. Given tourists’ varying preferences for Openness, Visual Entropy, and Aquatic Rate, scenic areas can be strategically divided into Cooling and Relaxation Zones (featuring shade and water elements) and Scenic Walking Zones (characterized by rich colors and open vistas). By implementing effective path planning and a well-designed signage system, visitors can be guided through these areas in an organized and seamless manner, enhancing both comfort and overall tourism experience. Second, at the management level, seasonal adaptation management can be implemented to dynamically adjust landscape strategies. High-temperature weather and recreational behaviors exhibit significant seasonal characteristics [100]. Scenic areas should timely adjust landscape maintenance and operational strategies according to environmental changes in different seasons. Implement seasonal landscape maintenance and upgrades by utilizing the relatively stable visitor flow during non-high-temperature seasons to enhance key scenic nodes, pathways, and vegetation. This proactive approach ensures that these elements provide both cooling effects and aesthetic appeal during high-temperature periods. Additionally, scenic areas can integrate local cultural characteristics across seasons by incorporating art installations, vibrant seasonal vegetation, or thematic exhibitions, fostering deeper sensory engagement and emotional connection between visitors and the natural environment.

During high-temperature periods in summer, strengthening the maintenance and irrigation of vegetation ensures healthy plant growth and visual appeal. Utilizing experimental data to identify areas where tourists’ emotions fluctuate significantly under high-temperature conditions allows for targeted optimization of landscape configurations. For instance, in areas with low vegetation coverage or poor plant quality, implementing ecological restoration or introducing heat-resistant plants can improve landscape quality and ecological functions. Additionally, prohibiting excessive construction development ensures positive visual experiences for tourists across different seasons and maintains ecosystem service functions [101]. Furthermore, enhancing landscape connectivity and implementing seasonal adaptation management strategies are not solely targeted at high-temperature environments. While the demand for shaded corridors and continuous water systems is more pronounced during hot seasons, these connectivity networks also play a crucial role in improving spatial fluidity, enriching biodiversity, and encouraging prolonged visitor engagement during cooler periods. By implementing seasonal maintenance for green corridors, water bodies, and key functional nodes in non-high-temperature seasons, scenic areas can sustain optimal environmental health while ensuring diverse visitor experiences across different temperature conditions. For low-vegetation or water-scarce areas, introducing heat-resistant plants and reinforcing waterbody shorelines can enhance shading, cooling, and recreational appeal in preparation for future heat waves. Additionally, leveraging the machine learning and sentiment analysis framework from this study, government agencies, management bodies, and research institutions can collaborate to track visitor feedback in real-time and develop data-driven dynamic optimization strategies. Scenic areas can pilot application scenarios, refine strategies based on successful cases, and eventually expand them into a replicable model that balances ecological conservation and visitor satisfaction. Furthermore, these best practices could be showcased for external visits or training programs, fostering broader adoption and knowledge dissemination.

### 4.3 Research contributions

This study integrates various machine learning methods with multimodal UGC data to explore the nonlinear impact of natural landscape perception indicators on tourist emotions under different temperature conditions. By providing a new perspective on ecotourism perception assessment, data collection, and analysis, the research aims to contribute to the sustainable development of global natural ecosystems and ecotourism.

(1) This study systematically examines how high-temperature and non-high-temperature conditions affect tourists’ perception of natural landscapes and emotional responses, offering a micro-level analysis of how tourists’ emotions shift based on landscape elements. It reveals significant changes in tourists’ perception of natural landscape indicators and visual quality under high-temperature conditions, addressing the limitation of previous research, which primarily focused on the macro-level impacts of climate conditions on tourism.
(2) The research adopts a multimodal data analysis approach, integrating UGC data, meteorological data, and advanced machine learning models such as LSTM-CNN, HRNet, and XGBoost, alongside geospatial analysis methods. A novel quantitative framework for assessing natural landscape perception is proposed. Additionally, by leveraging SHAP interpretability analysis, this study further uncovers the contributions and interactions of various landscape indicators on tourist emotions. Unlike traditional GIS-based and ethnographic studies, this research enhances SHAP analysis by incorporating boxplots, SHAP waterfall and force plots, significantly improving the granularity and precision of ecotourism perception studies.
(3) The findings have important practical implications for ecotourism planners, policymakers, and conservation managers. The proposed planning strategies offer insights into improving tourist comfort and emotional experiences in high-temperature environments. Furthermore, the data-driven framework serves as a monitoring tool for assessing climate change impacts on tourist destinations, providing scientific support for sustainable landscape planning. These insights help urban planners and decision-makers develop more effective ecotourism management strategies, promoting the long-term sustainability of natural landscapes.

### 4.4. Research limitations and further directions

Compared to traditional survey studies on natural landscape preferences and tourists’ emotional perceptions, this study utilizes UGC data combining images and texts, hotspot analysis, and machine learning methods to reveal the internal mechanisms by which natural landscape perception affects tourists’ emotions under different temperature conditions. However, there are certain limitations. First, this study was conducted in a representative city rich in natural ecological and tourism resources. Future research should be extended to other regions to assess the generalizability of the results, especially in cities with average or urgently needed ecotourism resources. Due to the randomness of UGC data, although we endeavored to collect data within the experimental scope, some UGC data and missing areas might not have been included in the analysis. This is an aspect that needs to be addressed in future studies. Additionally, future research should focus on longitudinal data collection using objective measures [102]. Second, due to privacy limitations of UGC data, we cannot accurately quantify the real emotions of people of different ages and cultural backgrounds toward the same scene. Factors that trigger human emotions are highly complex [103], including auditory and olfactory perceptions. The emotions perceived from text may differ from tourists’ experiences in natural landscapes [104]. Additionally, while Weibo and Ctrip provide a vast amount of data, they may still fail to capture the experiences of all tourists, particularly those who do not use such platforms. Platform specific biases and marketing influences could also introduce data gaps, potentially affecting the generalizability of the study’s findings. Moreover, the experiment used a binary classification method for quantifying perceived emotions, including positive and negative emotions. While this method facilitates comparison with other research results, it may oversimplify tourists’ complex emotional experiences.

In the future, provided that the dataset meets the requirements, more emotional classifications can be considered to explore the diverse emotional changes among residents. Finally, although this study extensively quantified and compared the impact of natural landscape elements on tourists’ emotions under high-temperature and non-high-temperature conditions, it did not fully consider the effects of seasonal changes and short-term weather fluctuations on tourist experiences. These temporal changes may influence the research results. Additionally, the classification of temperature conditions may have certain limitations. The definition of high-temperature and non-high-temperature conditions is primarily based on meteorological data thresholds, but tourists’ perception and adaptability to temperature may vary individually. Different tourists may have different feelings and reactions under the same temperature conditions. Future research should consider combining field surveys, in-depth interviews, and other methods to further enrich data sources and enhance the reliability and representativeness of the findings. Moreover, although this study proposes several landscape strategies based on experimental results, the effectiveness of these strategies during non-high-temperature periods remains unassessed due to the study’s time constraints. Future research should quantify the potential improvements these strategies may bring to the tourist experience, identify the most critical strategies for achieving positive outcomes, and evaluate the possible impacts of these strategies under different conditions.

## 5. Conclusion

This study focuses on Wuyishan City in China, an area with typical natural ecological resources. By utilizing UGC data combining images and texts and spatial analysis methods, along with machine learning models such as LSTM-CNN, Hrnet, and XGBoost, we compared tourists’ perceptions of natural landscapes and their emotional changes under high-temperature and non-high-temperature conditions. Through the SHAP method, we revealed the internal mechanisms by which natural landscape indicators affect tourists’ emotions under different temperature conditions and proposed sustainable optimization strategies for ecotourism natural landscapes based on the experimental results. The study found:

(1) Comparing the spatial autocorrelation analysis results under high-temperature and non-high-temperature conditions, we observed significant differences in the geographical distribution of tourists’ emotions. Under high-temperature conditions, the proportion of negative emotions significantly increased, and the spatial distribution was more dispersed (Z-score = -5.628), exhibiting clear spatial diffusion effects. This demonstrates the unique impact of high temperatures on tourists’ perceptions and emotions, prompting changes in photography preferences and destination choices.
(2) The SHAP interpretability analysis showed that under non-high-temperature conditions, the four most impactful indicators on tourists’ emotions were Color Complexity (0.73), Visual Entropy (0.71), Greenness (0.68), and Aquatic Rate (0.6). Under high-temperature conditions, the most influential indicators were Greenness (0.6), Openness (0.56), Visual Entropy (0.55), and Color Complexity (0.55). This indicates that tourists prioritize aesthetics and diversity under comfortable temperatures, while in high temperatures, they prefer landscape elements that enhance comfort, such as vegetation coverage and open spaces.
(3) The SHAP waterfall and force plot analyses revealed significant differences in the contributions of the Aquatic Rate under different temperatures, with a contribution of -0.27 under non-high-temperature conditions and 1.05 under high-temperature conditions. Furthermore, the overall benefit trend of key indicators under high-temperature conditions was more stable. This suggests that differences in tourists’ emotional perceptions are influenced not only by the physical characteristics of landscapes but also by their perception levels and adaptation methods. Planners should focus on differentiated conservation strategies and consider tourists’ environmental perceptions, especially under high-temperature conditions.

## Supporting information

S1 Data UGC crawl data. (ZIP)

## References

[1] Martinez-Juarez P, Chiabai A, Taylor T, Quiroga Gómez S. The impact of ecosystems on human health and well-being: A critical review. J Outdoor Recreation Tourism. 2015;10:63–9. doi: 10.1016/j.jort.2015.06.008

[2] Shang Y, Zhu L, Qian F, Xie Y. Role of green finance in renewable energy development in the tourism sector. Renewable Energy. 2023;206:890–6. doi: 10.1016/j.renene.2023.02.124

[3] Adnan MSG, Dewan A, Botje D, Shahid S, Hassan QK. Vulnerability of Australia to heatwaves: A systematic review on influencing factors, impacts, and mitigation options. Environ Res. 2022;213:113703. doi: 10.1016/j.envres.2022.113703 35716815

[4] Ali SB, Patnaik S. Thermal comfort in urban open spaces: Objective assessment and subjective perception study in tropical city of Bhopal, India. Urban Climate. 2018;24:954–67. doi: 10.1016/j.uclim.2017.11.006

[5] Chen X. Emotional Calculation Method of Rural Tourist Based on Improved SPCA-LSTM Algorithm. J Sensors. 2022;2022:1–9. doi: 10.1155/2022/3365498

[6] Chen Z, Yang H, Lin Y, Xie J, Xie Y, Ding Z. Exploring the association between the built environment and positive sentiments of tourists in traditional villages in Fuzhou, China. Ecol Informat. 2024;80:102465. doi: 10.1016/j.ecoinf.2024.102465

[7] Romanello M, Napoli C di, Green C, Kennard H, Lampard P, Scamman D, et al. The 2023 report of the Lancet Countdown on health and climate change: the imperative for a health-centred response in a world facing irreversible harms. Lancet. 2023;402(10419):2346–94. doi: 10.1016/S0140-6736(23)01859-7 37977174 PMC7616810

[8] Adger WN, Barnett J, Heath S, Jarillo S. Climate change affects multiple dimensions of well-being through impacts, information and policy responses. Nat Hum Behav. 2022;6(11):1465–73. doi: 10.1038/s41562-022-01467-8 36385175

[9] Patz JA. Global Climate Change and Emerging Infectious Diseases. JAMA. 1996;275(3):217. doi: 10.1001/jama.1996.03530270057032 8604175

[10] Patz JA, Campbell-Lendrum D, Holloway T, Foley JA. Impact of regional climate change on human health. Nature. 2005;438(7066):310–7. doi: 10.1038/nature04188 16292302

[11] Baylis P. Temperature and temperament: Evidence from Twitter. J Public Econ. 2020;184:104161. doi: 10.1016/j.jpubeco.2020.104161

[12] Berry HL, Bowen K, Kjellstrom T. Climate change and mental health: a causal pathways framework. Int J Public Health. 2010;55(2):123–32. doi: 10.1007/s00038-009-0112-0 20033251

[13] Keleş Özgenç E, Uzun O. Impacts of land use/land cover and climate change on landscape sensitivity in Tunca River sub-basin: Use in spatial planning and sectoral decision processes. J Environ Manage. 2024;363:121372. doi: 10.1016/j.jenvman.2024.121372 38843730

[14] Guo J, Li FY, Tuvshintogtokh I, Niu J, Li H, Shen B, et al. Past dynamics and future prediction of the impacts of land use cover change and climate change on landscape ecological risk across the Mongolian plateau. J Environ Manage. 2024;355:120365. doi: 10.1016/j.jenvman.2024.120365 38460328

[15] Rastandeh A, Jarchow M. Measuring the impacts of climate change on the spatial structure of grasslands in urban landscapes of North America. Urban Forestry Urban Greening. 2023;86:128000. doi: 10.1016/j.ufug.2023.128000

[16] Peng J, Tian L, Zhang Z, Zhao Y, Green SM, Quine TA, et al. Distinguishing the impacts of land use and climate change on ecosystem services in a karst landscape in China. Ecosys Services. 2020;46:101199. doi: 10.1016/j.ecoser.2020.101199

[17] Guo R, Guo F, Dong J, Wang Z, Zheng R, Zhang H. Finer-scale urban health risk assessment based on the interaction perspective of thermal radiation, human, activity, and space. Front Architectural Res. 2024;13(3):682–97. doi: 10.1016/j.foar.2024.02.002

[18] Zhang H, Wang Y, Guo F, Zhao J, Dong J, Zhu P. Factors influencing outdoor thermal comfort in a coastal park during the transition seasons in cold regions of China. Urban Climate. 2024;55:101856. doi: 10.1016/j.uclim.2024.101856

[19] Caldeira AM, Kastenholz E. It’s so hot: predicting climate change effects on urban tourists’ time–space experience. J Sustainable Tourism. 2018;26(9):1516–42. doi: 10.1080/09669582.2018.1478840

[20] Hunter RF, Cleland C, Cleary A, Droomers M, Wheeler BW, Sinnett D, et al. Environmental, health, wellbeing, social and equity effects of urban green space interventions: A meta-narrative evidence synthesis. Environ Int. 2019;130:104923. doi: 10.1016/j.envint.2019.104923 31228780

[21] Han J, Lee M, Hwang Y-S. Tourists’ Environmentally Responsible Behavior in Response to Climate Change and Tourist Experiences in Nature-Based Tourism. Sustainability. 2016;8(7):644. doi: 10.3390/su8070644

[22] Rupprecht C. Informal Urban Green Space: Residents’ Perception, Use, and Management Preferences across Four Major Japanese Shrinking Cities. Land. 2017;6(3):59. doi: 10.3390/land6030059

[23] Garvin EC, Cannuscio CC, Branas CC. Greening vacant lots to reduce violent crime: a randomised controlled trial. Inj Prev. 2013;19(3):198–203. doi: 10.1136/injuryprev-2012-040439 22871378 PMC3988203

[24] McCarthy LJ, Russo A. Exploring the role of nature-based typologies and stewardship schemes in enhancing urban green spaces: Citizen perceptions of landscape design scenarios and ecosystem services. J Environ Manage. 2023;346:118944. doi: 10.1016/j.jenvman.2023.118944 37738726

[25] Biernacka M, Kronenberg J, Łaszkiewicz E, Czembrowski P, Amini Parsa V, Sikorska D. Beyond urban parks: Mapping informal green spaces in an urban–peri-urban gradient. Land Use Policy. 2023;131:106746. doi: 10.1016/j.landusepol.2023.106746

[26] He D, Lu Y, Xie B, Helbich M. How greenway exposure reduces body weight: A natural experiment in China. Landscape and Urban Planning. 2022;226:104502. doi: 10.1016/j.landurbplan.2022.104502

[27] Anderson CE, Zimmerman A, Lewis S, Marmion J, Gustat J. Patterns of Cyclist and Pedestrian Street Crossing Behavior and Safety on an Urban Greenway. Int J Environ Res Public Health. 2019;16(2):201. doi: 10.3390/ijerph16020201 30642047 PMC6352138

[28] Paletto A, Becagli C, De Meo I. Aesthetic preferences for deadwood in forest landscape: A case study in Italy. J Environ Manage. 2022;311:114829. doi: 10.1016/j.jenvman.2022.114829 35287079

[29] Wei H, Hauer RJ, Sun Y, Meng L, Guo P. Emotional perceptions of people exposed to green and blue spaces in forest parks of cities at rapid urbanization regions of East China. Urban Forestry Urban Greening. 2022;78:127772. doi: 10.1016/j.ufug.2022.127772

[30] Chen Z, Ye C, Yang H, Ye P, Xie Y, Ding Z. Exploring the impact of seasonal forest landscapes on tourist emotions using Machine learning. Ecol Indicators. 2024;163:112115. doi: 10.1016/j.ecolind.2024.112115

[31] Guo F, Guo R, Zhang H, Dong J, Zhao J. A canopy shading-based approach to heat exposure risk mitigation in small squares. Urban Climate. 2023;49:101495. doi: 10.1016/j.uclim.2023.101495

[32] Zhang T, Zhang W, Meng H, Zhang Z. Analyzing Visitors’ Preferences and Evaluation of Satisfaction Based on Different Attributes, with Forest Trails in the Akasawa National Recreational Forest, Central Japan. Forests. 2019;10(5):431. doi: 10.3390/f10050431

[33] Liu Y, Ma J. Significant early end of the growing season of forest vegetation inside China’s protected areas. iScience. 2023;27(1):108652. doi: 10.1016/j.isci.2023.108652 38205259 PMC10776955

[34] Qian L, Guo J, Qiu H, Zheng C, Ren L. Exploring destination image of dark tourism via analyzing user generated photos: A deep learning approach. Tourism Management Perspectives. 2023;48:101147. doi: 10.1016/j.tmp.2023.101147

[35] Payntar ND, Hsiao W-L, Covey RA, Grauman K. Learning patterns of tourist movement and photography from geotagged photos at archaeological heritage sites in Cuzco, Peru. Tourism Management. 2021;82:104165. doi: 10.1016/j.tourman.2020.104165

[36] Kim D, Kang Y, Park Y, Kim N, Lee J. Understanding tourists’ urban images with geotagged photos using convolutional neural networks. Spat Inf Res. 2019;28(2):241–55. doi: 10.1007/s41324-019-00285-x

[37] Chen Z, Yang H, Ye P, Zhuang X, Zhang R, Xie Y, et al. How does the perception of informal green spaces in urban villages influence residents’ complaint Sentiments? a Machine learning analysis of Fuzhou City, China. Ecological Indicators. 2024;166:112376. doi: 10.1016/j.ecolind.2024.112376

[38] Zhang Y, Yan S, Liu J, Xu P. Popularity influence mechanism of creative industry parks: A semantic analysis based on social media data. Sustainable Cities Society. 2023;90:104384. doi: 10.1016/j.scs.2022.104384

[39] Zhang S-N, Li Y-Q, Ruan W-Q, Liu C-H. Would you enjoy virtual travel? The characteristics and causes of virtual tourists’ sentiment under the influence of the COVID-19 pandemic. Tourism Management. 2022;88:104429. doi: 10.1016/j.tourman.2021.104429

[40] Cao R, Zhu J, Tu W, Li Q, Cao J, Liu B, et al. Integrating Aerial and Street View Images for Urban Land Use Classification. Remote Sensing. 2018;10(10):1553. doi: 10.3390/rs10101553

[41] Zheng Y, Lin T, Hamm NAS, Liu J, Zhou T, Geng H, et al. Quantitative evaluation of urban green exposure and its impact on human health: A case study on the 3-30-300 green space rule. Sci Total Environ. 2024;924:171461. doi: 10.1016/j.scitotenv.2024.171461 38461976

[42] Biernacka M, Kronenberg J. Classification of institutional barriers affecting the availability, accessibility and attractiveness of urban green spaces. Urban Forestry Urban Greening. 2018;36:22–33. doi: 10.1016/j.ufug.2018.09.007

[43] Liu R, Yan X, Lin X, Sun Y, Zhang T, Xiao J. Urban spontaneous plant richness in response to the 2D/3D building and green space patterns in a highly urbanized area. Ecol Indicators. 2023;154:110852. doi: 10.1016/j.ecolind.2023.110852

[44] Yang W, Li Y, Liu Y, Fan P, Yue W. Environmental factors for outdoor jogging in Beijing: Insights from using explainable spatial machine learning and massive trajectory data. Landscape Urban Planning. 2024;243:104969. doi: 10.1016/j.landurbplan.2023.104969

[45] Liu Y, Lü Y, Fu B, Zhang X. Landscape pattern and ecosystem services are critical for protected areas’ contributions to sustainable development goals at regional scale. Sci Total Environ. 2023;881:163535. doi: 10.1016/j.scitotenv.2023.163535 37075999

[46] Dong Q, Sun X, Sheng J, Lei N. An experimental investigation on the damage mechanisms of red glutenite in the Mount Wuyi cultural and natural heritage site subject to acid rain and wet-dry cycles: a macro-to-micro approach. Herit Sci. 2024;12(1). doi: 10.1186/s40494-024-01393-0

[47] Fu W, Zhou B. Theme Exploration and Sentiment Analysis of Online Reviews of Wuyishan National Park. Land. 2024;13(5):629. doi: 10.3390/land13050629

[48] Lin X, D K, Guo H, J D, Ji X, W H. The causes of the abnormal increase of ozone in Fuzhou city under extreme high temperature. Ecol Environ. 2023;32:320. doi: 10.16258/j.cnki.1674-5906.2023.02.012

[49] Zheng X, Yang Z, Lu Y. Multidimensional Assessment of the Aesthetic Quality of Natural Landscapes in Mount Wuyi National Park, China. Land. 2024;13(10):1674. doi: 10.3390/land13101674

[50] Yang Z, Ren J, Zhang D. The Impact of the Establishment of the Mount Wuyi National Park on the Livelihood of Farmers. Agriculture. 2023;13(8):1619. doi: 10.3390/agriculture13081619

[51] Li S, Yang J, Cheng X, Liu Z. Multi-Agent Evolutionary Game Strategy for Ecotourism Development in National Parks: A Case Study of Wuyishan National Park. Forests. 2023;14(8):1590. doi: 10.3390/f14081590

[52] Yang ZF. Emergy Synthesis for Three Main Industries in Wuyishan City, China. J Env Inform. 2011;17(1):25–35. doi: 10.3808/jei.201100184

[53] Peng Y, Cui G, Li H, Wang N, Zheng X, Ding H, et al. Can CSR Strategy Classes Determined by StrateFy Explain the Species Dominance and Diversity of a Forest Community? Forests. 2024;15(8):1412. doi: 10.3390/f15081412

[54] Jeon JY, Jo HI. Effects of audio-visual interactions on soundscape and landscape perception and their influence on satisfaction with the urban environment. Building Environment. 2020;169:106544. doi: 10.1016/j.buildenv.2019.106544

[55] Mundher R, Abu Bakar S, Maulan S, Mohd Yusof MJ, Al-Sharaa A, Aziz A, et al. Aesthetic Quality Assessment of Landscapes as a Model for Urban Forest Areas: A Systematic Literature Review. Forests. 2022;13(7):991. doi: 10.3390/f13070991

[56] Li J, Nassauer JI, Webster NJ. Landscape elements affect public perception of nature-based solutions managed by smart systems. Landscape and Urban Planning. 2022;221:104355. doi: 10.1016/j.landurbplan.2022.104355

[57] Aboufazeli S, Jahani A, Farahpour M. Aesthetic quality modeling of the form of natural elements in the environment of urban parks. Evol Intel. 2022;17(1):327–38. doi: 10.1007/s12065-022-00768-1

[58] Qin X, Fang M, Yang D, Wangari VW. Quantitative evaluation of attraction intensity of highway landscape visual elements based on dynamic perception. Environmental Impact Assessment Review. 2023;100:107081. doi: 10.1016/j.eiar.2023.107081

[59] Yuan S, Browning MHEM, McAnirlin O, Sindelar K, Shin S, Drong G, et al. A virtual reality investigation of factors influencing landscape preferences: Natural elements, emotions, and media creation. Landscape Urban Plann. 2023;230:104616. doi: 10.1016/j.landurbplan.2022.104616

[60] Cai K, Huang W, Lin G. Bridging landscape preference and landscape design: A study on the preference and optimal combination of landscape elements based on conjoint analysis. Urban Forestry Urban Greening. 2022;73:127615. doi: 10.1016/j.ufug.2022.127615

[61] Ha J, Choi DH, Darling LE. Is the spatial distribution of urban green space associated with crime in Chicago?. Urban Forestry Urban Greening. 2024;95:128282. doi: 10.1016/j.ufug.2024.128282

[62] Brabyn L. Modelling landscape experience using “experions”. Appl Geogr. 2015;62:210–6. doi: 10.1016/j.apgeog.2015.04.021

[63] Chhetri P, Arrowsmith C. GIS-based Modelling of Recreational Potential of Nature-Based Tourist Destinations. Tourism Geographies. 2008;10(2):233–57. doi: 10.1080/14616680802000089

[64] Zhao X, Lu Y, Lin G. An integrated deep learning approach for assessing the visual qualities of built environments utilizing street view images. Engineering Applications Artificial Intelligence. 2024;130:107805. doi: 10.1016/j.engappai.2023.107805

[65] Xu T, Yao R, Du C, Li B. Outdoor thermal perception and heatwave adaptation effects in summer – A case study of a humid subtropical city in China. Urban Climate. 2023;52:101724. doi: 10.1016/j.uclim.2023.101724

[66] Yao R, Li Y, Du C, Li B. A ‘heart rate’-based model (PHSHR) for predicting personal heat stress in dynamic working environments. Building Environment. 2018;135:318–29. doi: 10.1016/j.buildenv.2018.03.014

[67] Qin Z, Lu B, Jing W, Yin Y, Zhang L, Wang X, et al. Creating comfortable outdoor environments: Understanding the intricate relationship between sound, humidity, and thermal comfort. Urban Climate. 2024;55:101967. doi: 10.1016/j.uclim.2024.101967

[68] He H, Sun R. Sentiment variations affected by urban temperature and landscape across China. Cities. 2024;149:104933. doi: 10.1016/j.cities.2024.104933

[69] Vo Q-H, Nguyen H-T, Le B, Nguyen M-L. Multi-channel LSTM-CNN model for Vietnamese sentiment analysis. In: 2017 9th International Conference on Knowledge and Systems Engineering (KSE). IEEE. 2017. 24–9. doi: 10.1109/kse.2017.8119429

[70] Sun K, Zhao Y, Jiang B, Cheng T, Xiao B, Liu D. High-resolution representations for labeling pixels and regions. 2019. doi: 10.48550/arXiv.1904.04514

[71] Wu C, Ye Y, Gao F, Ye X. Using street view images to examine the association between human perceptions of locale and urban vitality in Shenzhen, China. Sustainable Cities and Society. 2023;88:104291. doi: 10.1016/j.scs.2022.104291

[72] Stamps A. Entropy and visual diversity in the environment. J Archit Plan Res. 2004;21.

[73] Neale C, Griffiths A, Chalmin-Pui LS, Mendu S, Boukhechba M, Roe J. Color aesthetics: A transatlantic comparison of psychological and physiological impacts of warm and cool colors in garden landscapes. Wellbeing, Space Society. 2021;2:100038. doi: 10.1016/j.wss.2021.100038

[74] Zhou B, Xu S, Yang X. Computing the color complexity of images. 2015 12th International Conference on Fuzzy Systems and Knowledge Discovery (FSKD). Zhangjiajie, China: IEEE; 2015. pp. 1898–1902. doi: 10.1109/FSKD.2015.7382237

[75] Moran PAP. Notes on Continuous Stochastic Phenomena. Biometrika. 1950;37(1/2):17. doi: 10.2307/2332142 15420245

[76] Getis A, Ord JK. The Analysis of Spatial Association by Use of Distance Statistics. Geogr Anal. 1992;24(3):189–206. doi: 10.1111/j.1538-4632.1992.tb00261.x

[77] Peng D, Liang Z, Ding Y, Liang L, Zhai A, Zhang Y, et al. Spatial and temporal distribution characteristics and influencing factors of tourism eco-efficiency in the Yellow River Basin based on the geographical and temporal weighted regression model. PLoS One. 2024;19(2):e0295186. doi: 10.1371/journal.pone.0295186 38377110 PMC10878533

[78] Yan T, Jin H, Jin Y. The mediating role of emotion in the effects of landscape elements on thermal comfort: A laboratory study. Building Environment. 2023;233:110130. doi: 10.1016/j.buildenv.2023.110130

[79] Sinha J, Serin N. Exploring the impact of temperature perception and fear of missing out on distracted walking. Transportation Research Part F: Traffic Psychology and Behaviour. 2024;101:354–74. doi: 10.1016/j.trf.2023.12.014

[80] Song Y, Newman G, Huang X, Ye X. Factors influencing long-term city park visitations for mid-sized US cities: A big data study using smartphone user mobility. Sustainable Cities and Society. 2022;80:103815. doi: 10.1016/j.scs.2022.103815

[81] Kong L, Liu Z, Pan X, Wang Y, Guo X, Wu J. How do different types and landscape attributes of urban parks affect visitors’ positive emotions?. Landscape Urban Plann. 2022;226:104482. doi: 10.1016/j.landurbplan.2022.104482

[82] Tan X, Peng Y, Liu S, Liu P. Landscape pattern and ecotourism carrying capacity of Pan’an Lake wetland park in Xuzhou City, China. Desalination Water Treatment. 2020;188:288–96. doi: 10.5004/dwt.2020.25281

[83] Li J, Huang Z, Zhu Z, Ding G. Coexistence Perspectives: Exploring the impact of landscape features on aesthetic and recreational values in urban parks. Ecological Indicators. 2024;162:112043. doi: 10.1016/j.ecolind.2024.112043

[84] Rojas Bernal CL, Durosaiye IO, Hadjri K, Zabala Corredor SK, Segura Duran E, Cortés Prieto A. Neglected landscapes and green infrastructure: The case of the Limas Creek in Bogotá, Colombia. Geoforum. 2022;136:194–210. doi: 10.1016/j.geoforum.2022.09.010

[85] Huang C, Wei F, Qiu S, Cao X, Chen L, Xu J, et al. Interpreting regenerated post-industrial lands as green spaces: Comparing public perceptions of post-industrial landscapes using human factor design framework. Ecological Indicators. 2023;157:111282. doi: 10.1016/j.ecolind.2023.111282

[86] Yan T, Jin Y, Jin H. Combined effects of the visual-thermal environment on the subjective evaluation of urban pedestrian streets in severely cold regions of China. Building Environment. 2023;228:109895. doi: 10.1016/j.buildenv.2022.109895

[87] Dickinson JE, Filimonau V, Cherrett T, Davies N, Norgate S, Speed C. Understanding and governing sustainable tourism mobility. Routledge. 2014.

[88] Lau KK-L, Choi CY. The influence of perceived aesthetic and acoustic quality on outdoor thermal comfort in urban environment. Building Environment. 2021;206:108333. doi: 10.1016/j.buildenv.2021.108333

[89] Medeiros A, Fernandes C, Gonçalves JF, Farinha-Marques P, Martinho Da Silva I. How can landscape visual assessment inform landscape planning and management? – Alto Douro Wine region case study, Portugal. Appl Geography. 2024;164:103203. doi: 10.1016/j.apgeog.2024.103203

[90] Thorpert P, Englund J-E, Sang ÅO. Shades of green for living walls – experiences of color contrast and its implication for aesthetic and psychological benefits. Nature-Based Solutions. 2023;3:100067. doi: 10.1016/j.nbsj.2023.100067

[91] Li K, Liu M. Combined influence of multi-sensory comfort in winter open spaces and its association with environmental factors: Wuhan as a case study. Building Environment. 2024;248:111037. doi: 10.1016/j.buildenv.2023.111037

[92] de la Fuente de Val G. The effect of spontaneous wild vegetation on landscape preferences in urban green spaces. Urban Forestry Urban Greening. 2023;81:127863. doi: 10.1016/j.ufug.2023.127863

[93] Tabatabaie S, Litt JS, Muller BHF. Sidewalks, trees and shade matter: A visual landscape assessment approach to understanding people’s preferences for walking. Urban Forestry Urban Greening. 2023;84:127931. doi: 10.1016/j.ufug.2023.127931

[94] Wang L, Huang L, Cao W, Zhai J, Fan J. Assessing grassland cultural ecosystem services supply and demand for promoting the sustainable realization of grassland cultural values. Sci Total Environ. 2024;912:169255. doi: 10.1016/j.scitotenv.2023.169255 38092214

[95] Sun P, Liu P, Song Y. Seasonal variations in urban park characteristics and visitation patterns in Atlanta: A big data study using smartphone user mobility. Urban Forestry Urban Greening. 2024;91:128166. doi: 10.1016/j.ufug.2023.128166

[96] Wu R, Han Y, Chen S. Awe or excitement? The interaction effects of image emotion and scenic spot type on the perception of helpfulness. J Hospitality Tourism Management. 2024;58:76–84. doi: 10.1016/j.jhtm.2023.11.005

[97] Ding J, Tao Z, Hou M, Chen D, Wang L. A Comparative Study of Perceptions of Destination Image Based on Content Mining: Fengjing Ancient Town and Zhaojialou Ancient Town as Examples. Land. 2023;12(10):1954. doi: 10.3390/land12101954

[98] Sun H, Wei J, Han Q. Assessing land-use change and landscape connectivity under multiple green infrastructure conservation scenarios. Ecological Indicators. 2022;142:109236. doi: 10.1016/j.ecolind.2022.109236

[99] Zhang P, Fahey RT, Park S. The importance of current and potential tree canopy on urban vacant lots for landscape connectivity. Urban Forestry Urban Greening. 2024;94:128235. doi: 10.1016/j.ufug.2024.128235

[100] Shaaban K, Muley D, Elnashar D. Evaluating the effect of seasonal variations on walking behaviour in a hot weather country using logistic regression. Int J Urban Sciences. 2017;22(3):382–91. doi: 10.1080/12265934.2017.1403363

[101] Yang H, Yu J, Xu W, Wu Y, Lei X, Ye J, et al. Long-time series ecological environment quality monitoring and cause analysis in the Dianchi Lake Basin, China. Ecological Indicators. 2023;148:110084. doi: 10.1016/j.ecolind.2023.110084

[102] Patel J, Katapally TR, Khadilkar A, Bhawra J. The interplay between air pollution, built environment, and physical activity: Perceptions of children and youth in rural and urban India. Health Place. 2024;85:103167. doi: 10.1016/j.healthplace.2023.103167 38128264

[103] Ekman P. An argument for basic emotions. Cognition and Emotion. 1992;6(3–4):169–200. doi: 10.1080/02699939208411068

[104] Havinga I, Marcos D, Bogaart P, Tuia D, Hein L. Understanding the sentiment associated with cultural ecosystem services using images and text from social media. Ecosystem Services. 2024;65:101581. doi: 10.1016/j.ecoser.2023.101581

